# Molecular Characterization of the *14-3-3* Gene Family in *Brachypodium distachyon* L. Reveals High Evolutionary Conservation and Diverse Responses to Abiotic Stresses

**DOI:** 10.3389/fpls.2016.01099

**Published:** 2016-07-26

**Authors:** Hui Cao, Yuxing Xu, Linlin Yuan, Yanwei Bian, Lihui Wang, Shoumin Zhen, Yingkao Hu, Yueming Yan

**Affiliations:** Lab of Molecular Genetics and Proteomics, College of Life Science, Capital Normal UniversityBeijing, China

**Keywords:** *14-3-3* genes, *Brachypodium distachyon*, gene structures, phylogenetic relationships, gene duplication, abiotic stress, qRT-PCR

## Abstract

The *14-3-3* gene family identified in all eukaryotic organisms is involved in a wide range of biological processes, particularly in resistance to various abiotic stresses. Here, we performed the first comprehensive study on the molecular characterization, phylogenetics, and responses to various abiotic stresses of the *14-3-3* gene family in *Brachypodium distachyon* L. A total of seven *14-3-3* genes from *B. distachyon* and 120 from five main lineages among 12 species were identified, which were divided into five well-conserved subfamilies. The molecular structure analysis showed that the plant *14-3-3* gene family is highly evolutionarily conserved, although certain divergence had occurred in different subfamilies. The duplication event investigation revealed that segmental duplication seemed to be the predominant form by which the *14-3-3* gene family had expanded. Moreover, seven critical amino acids were detected, which may contribute to functional divergence. Expression profiling analysis showed that *BdGF14* genes were abundantly expressed in the roots, but showed low expression in the meristems. All seven *BdGF14* genes showed significant expression changes under various abiotic stresses, including heavy metal, phytohormone, osmotic, and temperature stresses, which might play important roles in responses to multiple abiotic stresses mainly through participating in ABA-dependent signaling and reactive oxygen species-mediated MAPK cascade signaling pathways. In particular, *BdGF14* genes generally showed upregulated expression in response to multiple stresses of high temperature, heavy metal, abscisic acid (ABA), and salicylic acid (SA), but downregulated expression under H_2_O_2_, NaCl, and polyethylene glycol (PEG) stresses. Meanwhile, dynamic transcriptional expression analysis of *BdGF14* genes under longer treatments with heavy metals (Cd^2+^, Cr^3+^, Cu^2+^, and Zn^2+^) and phytohormone (ABA) and recovery revealed two main expression trends in both roots and leaves: up-down and up-down-up expression from stress treatments to recovery. This study provides new insights into the structures and functions of plant *14-3-3* genes.

## Introduction

*14-3-3* proteins are a family of highly conserved acidic molecules of ~30 kDa, which were first identified in human brain and named by their particular elution and migration patterns on two-dimensional diethylaminoethyl-cellulose chromatography and starch gel electrophoresis (Moore et al., 1967). Further, studies revealed that, apart from their abundance in mammalian brain, *14-3-3* proteins are present in all eukaryotic organisms (Ferl, 1996). Such proteins are highly helical and generally dimeric in structure, including homo- and hetero-dimers, in which the monomer includes nine antiparallel α-helices, and the dimer creates a large negatively charged channel where the residues conserved among all of the isoforms can be found (Liu et al., 1995). Recent crystal structure analysis of *14-3-3* proteins has shown that the residues in the nine α-helices are highly conserved compared with other isoforms (Benzinger et al., 2005), while the C-terminal is the most variable region within the sequence, necessary for dimer formation and functional diversity (Jones et al., 1995).

Although a few *14-3-3* proteins interact with other proteins without a phosphorylated or even with a phosphorylated sequence, the majority of them specifically bind to phosphoserine- and/or phosphothreonine-containing motifs (Muslin et al., 1996). To date, three types of *14-3-3* unique binding motifs have been identified, including R[S/Φ][+]pSXP (mode I), RX[Φ/S][+]pSXP (mode II), and pS-X1-2-COOH (motif III), where Φ is an aromatic oraliphatic amino acid, + is a basic amino acid, pS is phosphoserine/phosphothreonine, and X is any type of amino acid (Yaffe et al., 1997). The phosphorylation of *14-3-3* proteins themselves on specific residues also has critical effects on their functional execution (Aitken, 2002; Wilson et al., 2016).

The sessile nature of plants results in them having to continuously monitor and adjust critical physiological processes to appropriately respond to changing external environmental conditions (Denison et al., 2011). The character of *14-3-3* proteins, binding other proteins to regulate their functions and/or changing the functional structure themselves, makes them ideally suited to regulate plants' internal physiological processes to respond to changing external environmental conditions (Aitken, 2006). The *14-3-3* protein family is involved in a wide range of biological processes in plants given their divergent functions, such as photosynthesis, primary metabolism, growth and cell division, hormone pathways, and abiotic and biotic stresses (de Boer et al., 2013). In particular, *14-3-3* proteins play key roles in the adverse stress responses of plants. For example, the overexpression of *Arabidopsis 14-3-3* λ in cotton directly revealed that *14-3-3* proteins play a major functional role in environmental stress responses (Yan et al., 2004). *14-3-3* proteins also have roles in the responses to biotic stress (Yang et al., 2009). In addition, *14-3-3* proteins have close and complex relationships with phytohormones, such as ABA, gibberellic acid (GA), and H_2_O_2_, which participates in hormone signaling pathways (Schoonheim et al., 2007; Cutler et al., 2010).

*Brachypodium distachyon* L. (2*n* = 10) is a wild annual grass endemic to the Mediterranean and Middle East, which belongs to the Pooideae subfamily in the grasses (Draper et al., 2001). It is promising as a model system of grasses, especially for economically valuable crops such as wheat and barley, because it has the physical and biological attributes necessary to serve as a model system, including small size, diploid accessions, a small genome (272 Mb), self-fertility, short generation time with a life cycle of < 4 months, a small stature but large seeds, and simple growth requirements (Draper et al., 2001; Vogel and Hill, 2008). The genome sequencing and annotation of *B. distachyon* 21 (Bd21) were completed in 2010, leading to the functional genome analysis available (International Brachypodium Initiative, 2010). To date, the plant *14-3-3* gene family has been widely investigated in a large number of green plants, such as *Arabidopsis* (Wu et al., 1997), rice (Chen et al., 2006), soybean (Li and Dhaubhadel, 2011), and *Medicago truncatula* (Qin et al., 2016). However, the structural features, phylogenetics, and functional properties of the *14-3-3* gene family in *B. distachyon* have not been reported so far.

In the present work, we performed the first comprehensive study on the molecular characterization, phylogenetic relationships, and abiotic stress responses of the *14-3-3* gene family in *B. distachyon* L. Our results provide new insights into the structures and functions of plant *14-3-3* genes.

## Materials and methods

### Sequences retrieval and identification of *14-3-3* genes

*14-3-3* gene families were identified from 13 species representing the plant lineage from unicellular green algae to multicellular plants. The search was performed using “*14-3-3*” as a keyword in TAIR database (https://www.arabidopsis.org/), and the Phytozome v10.3 database (http://www.phytozome.org). Thirteen *14-3-3* genes from *Arabidopsis thaliana* were first retrieved and then used as query sequences to run BLAST program against phytozome v10.3. The sequences obtained from corresponding plant-genome annotation resources were analyzed, in which the sequences up to *E* ≤ 1E–10 was selected as candidate sequences, and partial and redundant sequences were excluded manually. Sequences were collected from the following groups and species: the unicellular green algae *Chlamydomonas reinhardtii*; the moss *Physcomitrella patens*; the lycophyte *Selaginella moellendorffii*; the monocotyledonous angiosperms *Brachypodium distachyon, Oryza sativa, Triticum aestivum, Setaria italica, Zea mays*, and *Sorghum bicolor*; and the dicotyledonous angiosperms *Arabidopsis thaliana, Glycine max, Solanum lycopersicum*, and *Gossypium raimondii*. The *14-3-3* conserved domain of selected candidate sequences were further confirmed by the Simple Modular Architecture Research Tool (SMART, http://smart.embl-heidelberg.de/; Letunic et al., 2015) and Pfam (http://pfam.xfam.org/; Finn et al., 2014) database. The proteins up to *E* ≤ 1E–10, with methionine as the initial amino acid, and with amino acid sequence between 220 and 350 residues were selected as candidate proteins. Furthermore, sequences with partially conserved functional domains were further excluded, which is based on the search results of the SMART and Pfam (PF 00244). The protein sequences and the corresponding coding sequences and genomic sequences of the collected *14-3-3* genes were all obtained from phytozome database.

### Chromosomal locations and phylogenetic analysis

Location information of *14-3-3* genes on *B. distachyon* chromosome maps obtained from Phytozome v10.3 were mapped by MapInspect program and modified manually. Phylogenetic trees were constructed based on Bayesian inference using Markov Chain Monte Carlo (MCMC) method (Hall, 2005). Initially, full proteins were performed multiple sequence alignment based on MUSCLE program (http://www.ebi.ac.uk/Tools/msa/muscle/; Edgar, 2004). Bayesian inference phylogenetic construction was performed with MrBayes v 3.2 using GTR (General Time Reversible) model with Γ distributed rates (gamma-distributed rate variation) (Ronquist et al., 2012). Bayesian analysis included mcmc ngen = 10^6^ and samplefreq = 100. When the average standard deviation of split frequencies was below 0.01, the operation was terminated. After discarding the burn-in samples (first 25% of samples), the remaining data were used to generate a Bayesian tree, which was presented using software FigTree v1.4.2.

### Exon-intron structure, conserved motif, chemical character, and promoter analysis

The exon-intron structure and motif analysis of *14-3-3* genes were performed according to the programs described previously (Song et al., 2014) with minimal modifications, in which the maximum number of motifs = 10. The *14-3-3* protein pI/Mw were determined by the Compute pI/Mw tool (http://web.expasy.org/compute_pi/) (Bjellqvist et al., 1994). The 1500 bp upstream sequences as promoter regions were collected from Phytozome database, and they were then submitted to PlantCARE database (http://bioinformatics.psb.ugent.be/webtools/plantcare/html/; Lescot et al., 2002) to search their putative *cis*-acting elements.

### Analyses of duplication events, positive selection, functional divergence, and coevolution

The duplication events, positive selection, functional divergence, and coevolution analyses of *14-3-3* genes were conducted using multiple methods according to previous description (Song et al., 2014) with minimal modifications, of which the mean synonymous substitution rates (λ) used for the approximate date of the duplication event calculation are following: 6.5 × 10^−9^ for *Brachypodium* (Blanc and Wolfe, 2004), rice (Yu et al., 2005), and maize (Fan et al., 2014), 6.1 × 10^−9^ for soybean (Blanc and Wolfe, 2004), and 1.5 × 10^−8^ for Arabidopsis (Bowers et al., 2003). However, an accurate λ for *Solanum lycopersicum* and *Gossypium raimondii* was not found.

### Three-dimensional structure visualization of BdGF14 proteins

The structure of BdGF14 proteins was modeled by searching SWISS-MODEL database (http://swissmodel.expasy.org/) using the amino acid sequence (Biasini et al., 2014), which matched to the rice *14-3-3* protein (OsGF14c, PDB:3AXY), showing 235 from a total of 256 residues (Taoka et al., 2011). The Pymol software (Version 1.7.4 Schrödinger, LLC., http://pymol.org/) was applied to visualize the three-dimensional structure of BdGF14 proteins.

### Plant cultures and stress treatments

Before germination, the uniform seeds of standard diploid inbred line of Bd21 were sterilized with 75% alcohol and 15% sodium hypochlorite, and then washed three times with sterile water. After sterilization, the seeds were germinated on filter paper saturated with water in complete darkness condition at 26°C for 3 days. At the 4th day, seedlings were transferred to and grown in dedicated cultivation baskets with full-strength Hoagland solution [5 mM KNO_3_, 5 mM Ca(NO_3_)_2_, 2 mM MgSO_4_, 1 mM KH_2_PO_4_, 50 μM FeNa_2_(EDTA)_2_, 50 μM H_3_BO_3_, 10 μM MnC_12_, 0.8 μM ZnSO_4_, 0.4 μM CuSO_4_, and 0.02 μM (NH_4_)_6_MoO_24_.] in the greenhouse under a 16/8 h (light/dark) photocycle at 28/26°C (day/night) condition with relative humidity of 70%. The nutrient solution was changed every 3 days. Seedlings at three leaves stage were treated with the following conditions: salinity stress (200 mM NaCl), drought stress (20% (w/v) PEG 6000), heavy metal stress [300 μM Cd^2+^ (CdCl_2_), Cr^3+^ (CrCl_3_), Cu^2+^ (CuSO_4_) and Zn^2+^ (ZnSO_4_)], hormone stress (20 mM H_2_O_2_, 100μM ABA and SA), hot stress (42°C) and cold stress (4°C). Leaf and root samples of control seedlings were harvested at 0 h. Except for hot and cold stress, samples were collected at 2 h, the corresponding samples of treated seedlings were harvested at 6, 12, 24, 48 h and recover 48 h. Each sample was collected with three biological replicates. All samples were immediately stored at −80°C prior to use.

### Total mRNA extraction, qRT-PCR, and PCA analysis

Total mRNA was isolated from frozen samples using TRIzol Reagent (Invitrogen) according to the manufacturer's instructions. Genomic DNA removal and cDNA synthesis were conducted by using PrimeScript® RT reagent Kit with gDNA Eraser (TaKaRa). Gene-specific primers of each *14-3-3* gene in *B. distachyon* were designed using on-line tool Primer3Plus (http://www.bioinformatics.nl/cgi-bin/primer3plus/primer3plus.cgi; Untergasser et al., 2007). The primers were checked by blasting primer sequences in the NCBI database (http://www.ncbi.nlm.nih.gov/tools/primer-blast/index.cgi?LINK_LOC=BlastHome), and all primers were specifically consistent with the respective sequence of its target gene. The primer sequences for the qRT-PCR assays are listed in Table S1. The transcription level of each *14-3-3* gene in Bd21 were quantified with a CFX96 Real-Time PCR Detection System (Bio-Rad) using the intercalating dye SYBR-green following the 2(-Delta Delta C(T)) method (Livak and Schmittgen, 2001). The *B. distachyon* constitutively expressed S-adenosylmethionine decarboxylase gene (*SamDC*) was used as a reference for normalization (Hong et al., 2008). qRT-PCR was performed in a 20 μL volume reaction system containing 10 μL 2 × SYBR® Premix ExTaq® (TaKaRa), 2 μL 10-fold diluted cDNA, 0.15 μL of each gene-specific primer and 7.7 μL ddH2O. The PCR conditions were as follows: 95°C for 3 min, 40 cycles at 95°C for 20 s, 61°C for 15 s, and 72°C for 10 s. Triplicates for each PCR reaction and at least three biological replicates were performed for each gene. The qRT-PCR efficiency was determined by five serial 10-fold dilutions of cDNA. The optimal performance was conducted, in which the PCR amplification efficiency (E) of 95–105% and correlation coefficient (*R*^2^) of 0.995–0.999 were controlled (Figure S1).

The hierarchical cluster analysis of qRT-PCR results was performed using Cluster 3.0 according to the method described by Eisen et al. (1998) with minor modifications. In particular, the gene expression level at 0 h was defined as 1, the gene expression level at the remaining time periods was changed according to the original ratios. Subsequently, the relative ratios of gene expression were conducted Log2 transforming, and then the Euclidean distance similarity metric was used to define the similarity and the hierarchical clusters were assembled using the complete-linkage clustering method.

Principal Component Analysis (PCA) is a method that focuses on finding the main variations and revealing hidden structures present in the data set (Kristiansen et al., 2010). In the present study, coefficient and KMO and Bartlett's test of sphericity were used for dimension reduction analysis and the results were shown in the loading plot and scatter plot, respectively. PCA was performed using SPSS 19.0 software, and the loading plot and scatter plot of PCA was calculated and displayed with the average expression level of each genes.

## Results

### Genome-wide identification of the *14-3-3* gene family in *B. distachyon* and 12 other plant species

To explore the molecular characteristics and phylogenetic relationships of the *14-3-3* gene family in *B. distachyon* and other main plant species, we initially downloaded 13 known *Arabidopsis 14-3-3* protein sequences from the TAIR database (https://www.arabidopsis.org/) and the Phytozome v10.3 database (http://www.phytozome.org), which were subsequently used as queries for searches of the Phytozome v10.3 database. The BLASTP search results were further checked using the online tools SMART and Pfam to confirm the presence of conserved *14-3-3* domains, and redundant and partial sequences were removed manually. Finally, a total of seven *14-3-3* genes from *B. distachyon* and 120 from 12 other plant species were obtained, including 2 from *Chlamydomonas reinhardtii*, 10 from *Physcomitrella patens*, 4 from *Selaginella moellendorffii*, 7 from *Oryza sativa*, 10 from *Triticum aestivum*, 9 from *Setaria italica*, 12 from *Zea mays*, 5 from *Sorghum bicolor*, 13 from *Arabidopsis thaliana*, 18 from *Glycine max*, 13 from *Solanum lycopersicum*, and 17 from *Gossypium raimondii* (Figure S2). Based on the nomenclature suggestions of rice *14-3-3* genes, each gene except for those from *Arabidopsis* was named as follows: the first two letters correspond to the species (for example, Os: *Oryza sativa*), followed by the family designation (GF14) and a letter as shown. Basic information on these *14-3-3* genes (including gene name, loci, protein length, intron number, predicted isoelectric point (PI), and molecular weight) was shown in Table S2, and their conserved motifs identified by SMART and Pfam were listed in Table S3. The encoding proteins of *14-3-3* genes ranged from 230 to 342 amino acids (aa) in length, and the majority of them were about 260 aa. The PI and molecular weight (MW) ranged from 4.61 to 6.26 and 25.85 to 38.13 kDa, respectively.

**Figure 1:**
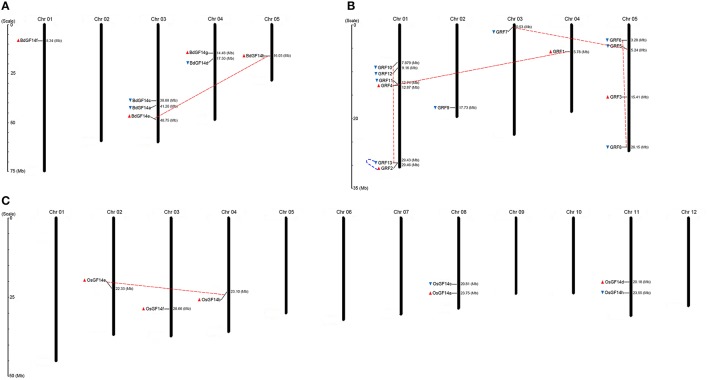
**Chromosomal distribution map of ***14-3-3*** genes in ***B. distachyon***, Arabidopsis and rice. (A)** *B. distachyon*. **(B)** Arabidopsis. **(C)** Rice. The chromosome numbers are indicated at the top of each bar, while the size of a chromosome is indicated by its relative length. The sea blue triangle and red triangle indicate the transcriptional direction of genes, sea blue (5′ to 3′) and red (3′ to 5′). The dashed lines with sea blue color represent tandem duplicated genes, whereas the dashed lines with red color represent segmentally duplicated genes.

*14-3-3* genes of *B. distachyon*, rice, and *Arabidopsis* were mapped to their chromosomes using the MapInspect program (Figure 1). In *B. distachyon*, the predicted seven *BdGF14* genes were located on four chromosomes, in which *BdGF14f* was located on chromosome 01 while three *BdGF14* genes (*BdGF14a, c*, and *e*) were on chromosome 03. The remaining three genes were distributed on chromosomes 04 (*BdGF14d* and *g*) and 05 (*BdGF14b*). In *Arabidopsis*, chromosome 01 had six *14-3-3* genes (*GRF2, 4, 10, 11, 12*, and *13*), as the largest group of *14-3-3* genes, followed by chromosome 05 with four genes (*GRF3, 5, 6*, and *8*). The last three genes (*GRF9, 7*, and *1*) were distributed on the chromosome 02, 03, and 04, respectively. Similarly, rice *OsGF14a* and *c* were located on chromosome 08 and *OsGF14d* and *h* on chromosome 11, while *OsGF14e, f* and *b* were located on the chromosome 02, 03, and 04, respectively. These results suggest that *14-3-3* genes have no obvious chromosomal preferences and that they are more likely to be clustered together in certain chromosomal regions.

### Phylogenetic and structural analyses of *14-3-3* genes

**Figure 2:**
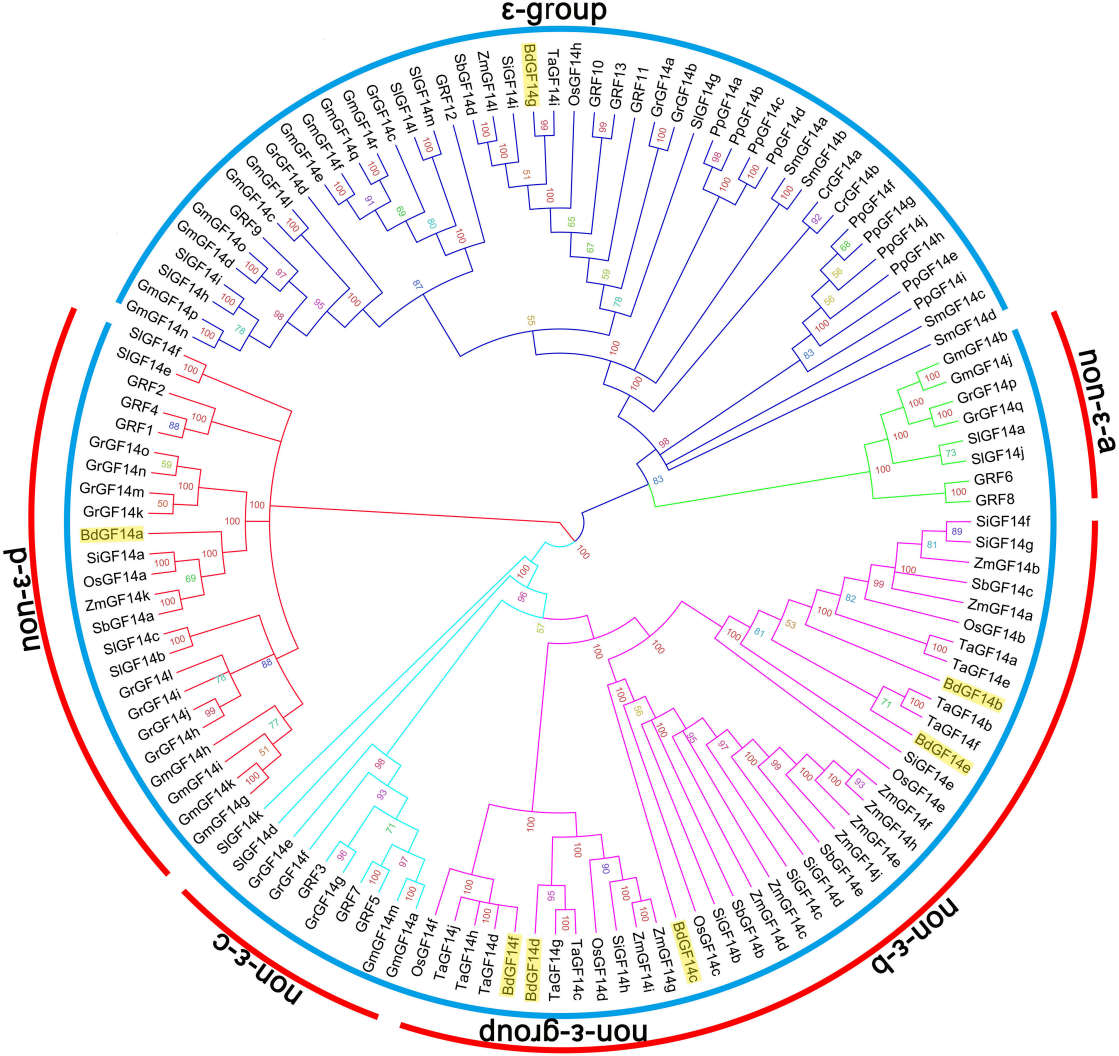
**Phylogenetic relationships of plant ***14-3-3*** genes**. A total of 127 complete protein sequences of the corresponding *14-3-3* genes obtained from 13 species from unicellular green algae to multicellular plants were aligned with MUSCLE program, and the phylogenetic tree was constructed based on Bayesian inference using Markov Chain Monte Carlo (MCMC) methods. The sky blue arcs indicate subgroups of *14-3-3* genes, while the red arcs indicate subfamilies of non-ε-group *14-3-3* genes. The *14-3-3* genes from *B. distachyon* are indicated by filled yellow rectangle.

To investigate the evolutionary relationships of *14-3-3* genes within multiple plant species, the full amino acid sequences of 127 identified proteins were used for multiple sequence alignment using the Multiple Sequence Comparison by Log-Expectation (MUSCLE) program. An unrooted phylogenetic tree was constructed using the Markov Chain Monte Carlo (MCMC) method based on Bayesian inference (Hall, 2005), as shown in Figure 2. To further confirm the topology of the phylogenetic tree, an unrooted neighbor-joining (N-J) phylogenetic tree was constructed, which exhibited similar topology to the Bayesian tree with only minor modifications (data not shown). According to the topology, the phylogenetic tree was separated into two distinct subgroups (ε-group and non-ε-group). Similarly, the non-ε-group could be further divided into four subgroups, named the non-ε-a subgroup, non-ε-b subgroup, non-ε-c subgroup, and non-ε-d subgroup. Among these five subgroups, ε-group was the largest, containing 46 *14-3-3* gene members, followed by the non-ε-b subgroup (39 members), non-ε-d subgroup (24 members), non-ε-c subgroup (10 members), and non-ε-a subgroup (8 members).

**Figure 3:**
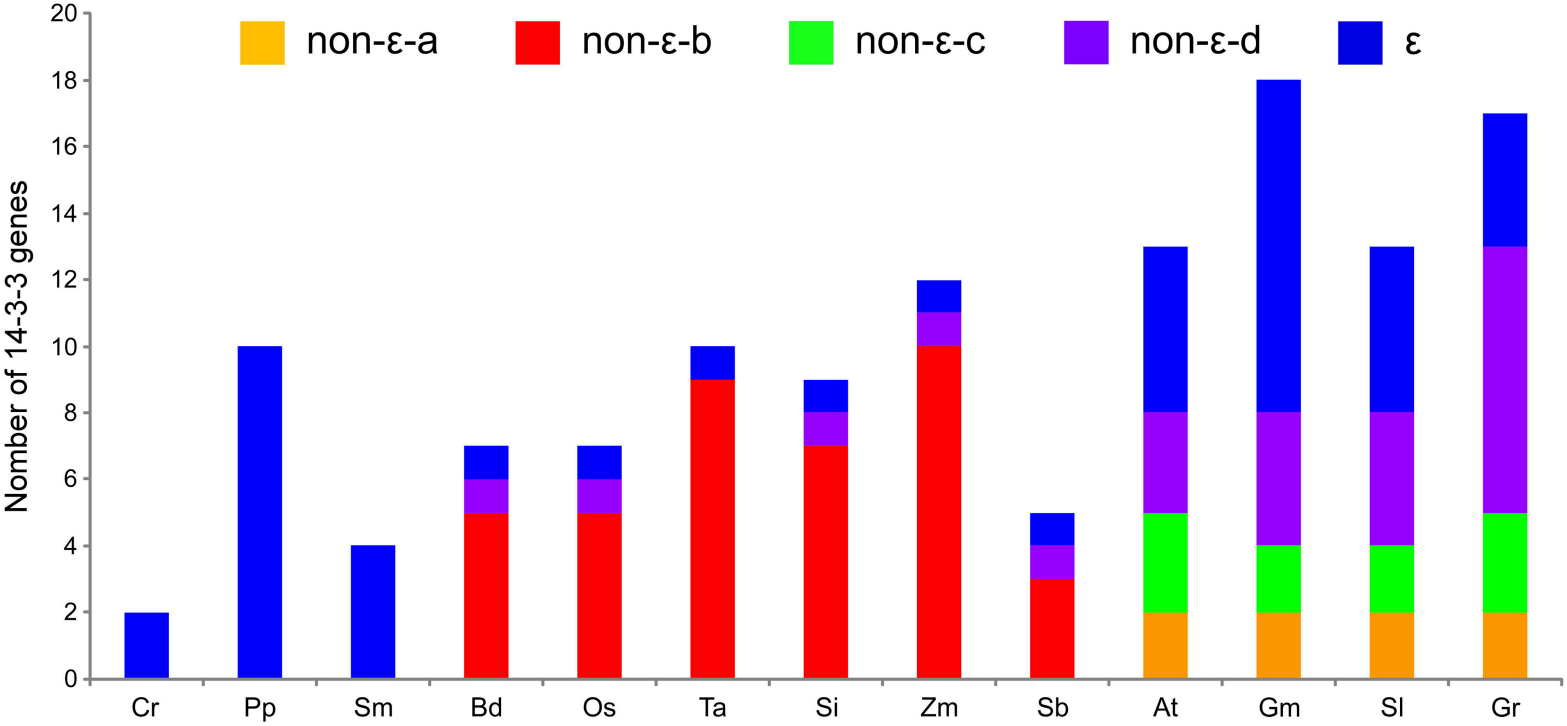
**The distribution of ***14-3-3*** genes based on subgroups in individual species**. Cr, Chlamydomonas reinhardtii; Pp, Physcomitrella patens; Sm, Selaginella moellendorffii; Bd, Brachypodium distachyon; Os, Oryza sativa; Ta, Triticum aestivum; Si, Setaria italica; Zm, Zea mays; Sb, Sorghum bicolor; At, Arabidopsis thaliana; Gm, Glycine max; Sl, Solanum lycopersicum; Gr, Gossypium raimondii.

In detail, the distribution of *14-3-3* genes in different individual species had significant differences, as well as unique characters in each subgroup (Figure 3). *14-3-3* genes identified from lower plant species, including the unicellular green algae (Cr), moss (Pp), and lycophyte (Sm), were all present in the ε-group, while the non-ε-b subgroup only contained monocotyledon genes (Bd, Os, Ta, Si, Zm, and Sb), which included the majority of monocot *14-3-3* genes. Similarly, the non-ε-a and non-ε-c subgroups only contained the dicot genes (At, Gm, Sl, and Gr). On the other hand, the distribution among the same lineage showed high consistency. For *B. distachyon* and rice in the monocots, for example, *BdGF14g* belonged to the ε-group, while *BdGF14a* to the non-ε-d subgroup and *BdGF14b-f* to the non-ε-b subgroup (highlighted by green rectangles in (Figure 2). The phylogenetic tree showed that *14-3-3* genes in rice had almost the same distribution pattern as those in *B. distachyon*, in which the non-ε-b subgroup contained *OsGF14b-f*, and *OsGF14a* was separately placed in the non-ε-d subgroup. Among the dicots, the distribution patterns of *14-3-3* genes were also similar.

The exon-intron organization of *14-3-3* genes in all selected plant lineages was analyzed by comparing the predicted coding sequences (CDSs) and their corresponding genomic sequences using the online tool Gene Structure Display Server (http://gsds.cbi.pku.edu.cn/). A detailed schematic diagram of the relative length of introns and conservation of the corresponding exon sequences within individual *14-3-3* gene paralog was shown in Figure S3, which was grouped and listed according to the phylogenetic tree construction. The results showed that gene members of the same subgroup possessed similar exon-intron structures. *14-3-3* genes generally had one to six introns, and most of them had three to five (Figure S4). There were no significant differences in the number of introns among individual genes within the same subgroup, while considerable differences were present in different subgroups. The ε-group had more introns than the non-ε-group. In particular, three subgroups, non-ε-a, non-ε-c, and non-ε-d subgroups, had a high percentage of *14-3-3* genes with three introns, up to 62.5, 90.0, and 83.3%, respectively. However, in the non-ε-b subgroup, those with four introns accounted for 89.7%. As the largest subgroup, the ε-group contained 29 genes (63.0%) with five introns, followed by those with three or four introns (15%) (Figure S4A). For the individual species, *14-3-3* genes had multiple introns, but the *14-3-3* genes in the monocots tended to have four introns, while those in the dicots had three or five (Figure S4B).

Conserved protein motif analysis of *14-3-3* genes was conducted using the Multiple Em for Motif Elicitation (MEME) program (http://meme-suite.org/). In total, 10 conserved motifs were detected from 127 *14-3-3* genes, as shown in Figure S5; the order and number of motifs in individual genes were shown in Figure S6. The number of motifs contained in *14-3-3* genes was primarily seven or eight (Figure S7A). Similarly to the introns in *14-3-3* genes, the number of conserved motifs was highly consistent within the subgroup, but considerable differences were present in different subgroups. All *14-3-3* genes in the non-ε-a subgroup had seven motifs, while the dominant number of motifs in the non-ε-b, non-ε-c, and non-ε-d subgroups was eight, accounting for 89.7, 90.0, and 79.2%, respectively. The ε-group, different from the other four non-ε groups, primarily contained seven or eight motifs, reaching 39.1 and 56.5% of the total, respectively. For individual species, there were similar patterns in the number of conserved motifs, although there were significant differences in different lineages. Most of the *14-3-3* genes in the lower land plants had seven motifs, but those in monocot and dicot species had eight (Figure S7B). These characteristics were highly consistent with the number of introns.

### Duplication events in the *14-3-3* gene family

In this study, we investigated the duplication events in the 1*4-3-3* gene family. The results showed that 14.3% (1 of 7) of *14-3-3* genes were derived from segmental duplication in *B. distachyon*, the same as in rice, but this rate was much lower than in *Arabidopsis* (30.8%, 4 of 13) (Figure 1). Among 13 plant species, soybean (Gm) had been most active in undergoing *14-3-3* gene duplication events, in which 55.6% (10 of 18) were derived from segmental duplication presented in 16 pairs of segmental duplication genes (Table 1). Among the lineages, dicots had 23 genes (37.7%) resulting from segmental duplication, corresponding to 32 pairs of segmental duplication genes, which is a much higher rate than in monocots (10.0%). The lower plant species had not undergone segmental duplication events. In addition, the segmental duplication events in monocots had only occurred in the non-ε-b subgroup, while those in dicots were separated among the remaining four subgroups.

**Table 1:** **Estimates of the dates for the segmental duplication events of ***14-3-3*** gene family**.

**Gene pairs**		**KS (means ± s.d.)**	**Estimated time (mya)**	**GWD (mya)**	**References**
*BdGF14b*	*BdGF14e*	0.593 ± 0.140	45.6	56–72	International Brachypodium Initiative, 2010
*OsGF14b*	*OsGF14e*	0.612 ± 0.228	47.0	30–40	Vandepoele et al., 2003
*ZmGF14a*	*ZmGF14b*	0.270 ± 0.000	20.8	5–12, 70	Schnable et al., 2009
*ZmGF14c*	*ZmGF14d*	0.200 ± 0.020	15.4		
*ZmGF14g*	*ZmGF14i*	0.330 ± 0.120	25.4		
*GRF10*	*GRF13*	0.811 ± 0.116	27.0	28–48	Ermolaeva et al., 2003
*GRF6*	*GRF8*	0.732 ± 0.144	24.4		
*GRF5*	*GRF7*	0.656 ± 0.136	21.9		
*GRF1*	*GRF4*	0.700 ± 0.000	23.3		
*GmGF14a*	*GmGF14m*	0.182 ± 0.166	14.9	13, 59	Schmutz et al., 2010
*GmGF14c*	*GmGF14l*	0.138 ± 0.048	11.3		
*GmGF14d*	*GmGF14o*	0.147 ± 0.049	12.0		
*GmGF14e*	*GmGF14f*	0.124 ± 0.036	10.2		
*GmGF14e*	*GmGF14q*	0.620 ± 0.035	50.8		
*GmGF14e*	*GmGF14r*	0.595 ± 0.060	48.8		
*GmGF14f*	*GmGF14q*	0.638 ± 0.025	52.3		
*GmGF14f*	*GmGF14r*	0.715 ± 0.147	58.6		
*GmGF14g*	*GmGF14i*	0.613 ± 0.203	50.2		
*GmGF14g*	*GmGF14h*	0.618 ± 0.141	50.7		
*GmGF14g*	*GmGF14k*	0.123 ± 0.048	10.1		
*GmGF14i*	*GmGF14h*	0.172 ± 0.055	14.1		
*GmGF14h*	*GmGF14k*	0.450 ± 0.020	36.9		
*GmGF14i*	*GmGF14k*	0.590 ± 0.137	48.4		
*GmGF14n*	*GmGF14p*	0.135 ± 0.060	11.1		
*GmGF14q*	*GmGF14r*	0.143 ± 0.048	11.7		
*SlGF14d*	*SlGF14k*	0.470 ± 0.000	^*^	52–91	Tomato Genome Consortium, 2012
*SlGF14l*	*SlGF14m*	0.628 ± 0.080	^*^		
*SlGF14h*	*SlGF14i*	0.810 ± 0.120	^*^		
*GrGF14a*	*GrGF14b*	0.413 ± 0.096	^*^	60	Paterson et al., 2012
*GrGF14h*	*GrGF14l*	0.593 ± 0.222	^*^		
*GrGF14h*	*GrGF14i*	0.607 ± 0.131	^*^		
*GrGF14k*	*GrGF14m*	0.445 ± 0.105	^*^		
*GrGF14k*	*GrGF14o*	0.455 ± 0.105	^*^		
*GrGF14m*	*GrGF14o*	0.570 ± 0.227	^*^		
*GrGF14m*	*GrGF14n*	0.630 ± 0.110	^*^		
*GrGF14n*	*GrGF14o*	0.460 ± 0.000	^*^		
*GrGF14p*	*GrGF14q*	0.745 ± 0.195	^*^		

* *represents the unknown data*.

Tandem duplication events often generate multiple members of one family within the same or neighboring intergenic regions. Interestingly, the tandem duplication events of *14-3-3* genes were extremely rare compared with the segmental duplication events and only four pairs of tandem duplication genes (*PpGF14f/PpGF14g, PpGF14i/PpGF14j, GRF13/GRF2*, and *GmGF14m/GmGF14n*) were found.

To estimate the approximate ages of segmental duplication events, the synonymous base substitution rates (*Ks*-values) were used as a proxy for time. The results showed that only one pair of segmental duplication genes were found in *B. distachyon* and rice, while maize, *Arabidopsis*, soy bean, tomato, and cotton had three, four, sixteen, three, and nine pairs, respectively (Table 1). The segmental duplication genes in *B. distachyon* were estimated to have originated ~45.6 million years ago (MYA), roughly consistent with the occurrence of large-scale duplications 56–72 MYA (International Brachypodium Initiative, 2010). In rice, the estimated time of emergence of the candidate segmental duplication genes was 47.0 MYA, also roughly consistent with the occurrence of large-scale duplications in rice 30–40 MYA (Vandepoele et al., 2003). Similarly, the estimated times of segmental duplication events for the remaining segmental duplication genes identified, except for those in tomato and cotton, roughly matched their genome-wide duplication(GWD; Table 1).

### Functional divergence in the *14-3-3* gene family

The Type I and Type II functional divergences of gene clusters in the *14-3-3* gene family were estimated through a posterior analysis using the DIVERGE v3.0 program to investigate whether amino acid substitutions caused functional diversification, which was based on the N-J trees (data not shown).

For Type I functional divergence (θI), the estimated likelihood ratio statistic (LRT) values of the 10 pairs of *14-3-3* gene subfamilies ranged from 0.000 to 6.480 (Table 2). Especially for the subfamily pairs of non-ε-a/non-ε-c, non-ε-a/non-ε-d, and non-ε-a/non-ε-b, they were ranked in the first three positions among the 10 subfamily pairs, with LRT values up to 6.480, 5.426, and 2.802, respectively. Subsequently, we analyzed whether Type II functional divergence (θII) occurred among the pairs of *14-3-3* gene subfamilies. The results showed that Type II (θII) coefficients between five subfamily pairs, namely, ε/non-ε-a, ε/non-ε-b, ε/non-ε-c, ε/non-ε-d, and non-ε-a/non-ε-d, were all significantly greater than zero, indicating that there were significant changes in amino acid properties among these subfamilies. In contrast, the other five pairs of *14-3-3* gene subfamilies (non-ε-a/non-ε-b, non-ε-a/non-ε-c, non-ε-b/non-ε-c, non-ε-b/non-ε-c, and non-ε-c/non-ε-d) had coefficients of < 0, suggesting that no significant changes had occurred in amino acid properties between these subfamilies (Table 2).

**Table 2:** **Functional divergence between subfamilies of the ***14-3-3*** gene family**.

**Group1**	**Group2**	**Type-I**			**Type-II**	
		**θI ± s.e**.	**LRT**	**Qk > 0.99**	**θII ± s.e**.	**Qk > 0.99**
ε	non-ε-a	0.27 ± 0.022	0.000	0	0.202 ± 0.137	45
ε	non-ε-b	0.001 ± 0.022	0.000	0	0.270 ± 0.134	0
ε	non-ε-c	0.001 ± 0.022	0.000	0	0.194 ± 0.136	46
ε	non-ε-d	0.060 ± 0.061	0.957	0	0.284 ± 0.126	0
non-ε-a	non-ε-b	0.186 ± 0.111	2.802	0	0.052 ± 0.064	5
non-ε-a	non-ε-c	0.278 ± 0.110	6.480^*^	0	0.045 ± 0.050	4
non-ε-a	non-ε-d	0.150 ± 0.065	5.426^*^	0	0.063 ± 0.058	5
non-ε-b	non-ε-c	0.121 ± 0.118	1.063	0	−0.010 ± 0.058	0
non−ε−b	non−ε−d	0.042 ± 0.073	0.337	0	−0.010 ± 0.065	0
non−ε−c	non−ε−d	0.006 ± 0.082	0.005	0	−0.022 ± 0.053	0

θI and θII, the coefficients of Type-I and Type-II functional divergence; LRT, Likelihood Ratio Statistic, for

* *p < 0.05; Qk, posterior probability*.

To further identify the critical amino acid sites for functional divergence in *14-3-3* gene subfamilies, the posterior probability (Qk) of divergence was calculated for each site. Type I and Type II functional divergence-related residues between the specified subgroups with Qk < 0.99 were excluded to reduce false positives. No critical amino acid sites were found for Type I functional divergence in any *14-3-3* subfamily pairs, as shown in Table 2 and Table S4. In contrast, Type II functional divergence-related residues were much more numerous than those of Type I. In particular, 45, 46, and 5 amino acid sites critical for Type II functional divergence were identified in ε/non-ε-a, ε/non-ε-c, and non-ε-a/non-ε-d subfamily pairs, respectively. These results suggested that functional divergence in these groups was primarily attributable to rapid changes in amino acid physiochemical properties, not to changes in evolutionary rate. The non-ε-a/non-ε-b and non-ε-a/non-ε-c subfamily pairs had five and four critical amino acid sites, respectively. However, these two subfamily pairs had a θII coefficient < 0, suggesting that both subfamily pairs may also have undergone functional divergence during evolutionary history, but not at a significant level. For the remaining five gene subfamily pairs (ε/non-ε-b, ε/non-ε-d, non-ε-b/non-ε-c, non-ε-b/non-ε-d, and non-ε-c/non-ε-d), no critical amino acid sites were identified, indicating that functional divergence among these subfamily pairs was not significant.

### Positive selection and coevolution analysis of the *14-3-3* gene family

To assume variable selective pressure among sites but no variation among branches in the phylogeny, a site-specific likelihood model was employed to the *14-3-3* gene family of the selected 13 species. Two pairs of models were applied in this analysis, forming two LRTs: M0 (one ratio) and M3 (discrete), and M7 (beta) and M8 (beta and ω). However, in the results, no amino acid sites were identified to be under positive selection (Table S5), suggesting the lack of selective pressure among the *14-3-3* gene family.

According to the negative results from the site-specific likelihood model, we further conducted a branch-site model analysis of the *14-3-3* gene family to test the positive selection effect at individual sites in different subfamilies. From the results, two critical positive selection sites were identified in the ε subfamily (1M and 148S, *P* < 0.01) (Table 3). Apart from the ε subfamily, two critical positive selection sites 2S and 95S, and 96K and 108H, were also found in each of the non-ε-a and non-ε-d subfamilies, respectively. The remaining two subfamilies (non-ε-b and non-ε-c) had no positive selection sites and had undergone less selective pressure. Furthermore, protein sequences (CAPS) were used for coevolution analysis, and three amino acids showed coevolution among the 13 plant species, namely, 236L, 237T, and 238E (Table S6).

**Table 3:** **Parameters estimation and likelihood ratio tests for the branch-site and free-ratio models among ***14-3-3*** genes**.

**Cluster**	**Model**	**np^a^**	**lnL**	**2⊿l**	**Positive selected sites^b^**
ε	Model A-null	255	−27619.537638		Not allowed
	Model A	256	−27613.788734	11.50^**^	1M, 148S
non-ε-a	Model A-null	255	−27621.004255		Not allowed
	Model A	256	−27619.359293	3.39	2S, 95S
non-ε-b	Model A-null	255	−27627.122669		Not allowed
	Model A	256	−27627.122670	0.00	none
non-ε-c	Model A-null	255	−27627.122670		Not allowed
	Model A	256	−27627.122670	0.00	none
non-ε-d	Model A-null	255	−27617.430951		Not allowed
	Model A	256	−27616.779011	1.30	96K, 108H

p < 0.01 are marked by ^**^.

a *Number of parameters in the ω distribution*.

b *The numbers of Positive-selection sites are inferred at posterior probabilities >95%*.

*Red color marked amino acids were identified in both Type-II functional divergence analysis and positive selection analysis*.

### Three-dimensional structure prediction of *14-3-3* proteins and identification of critical amino acid sites

**Figure 4:**
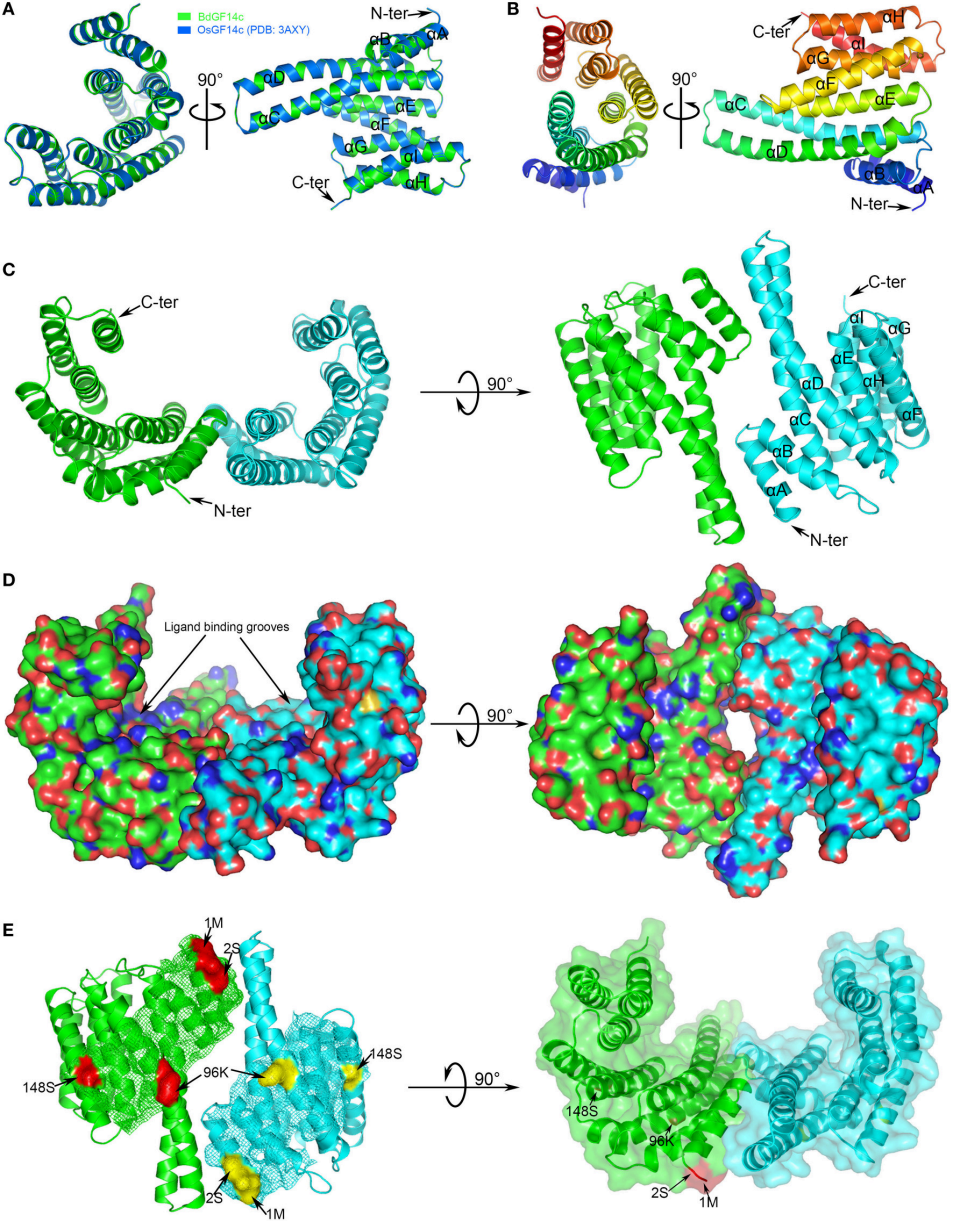
**Three-dimensional structure of ***B. distachyon 14-3-3*** protein BdGF14c. (A)** Structural comparison of BdGF14c (green) and OsGF14c (PDB: 2o98B, marine). The N- and C-terminals are indicated, the helices are labeled αA-αI. **(B)** Structure of BdGF14c monomer. **(C)** Structure of BdGF14c homodimer. Monomer 01 labeled green color, monomer 02 labeled cyan color. **(D)** Electrostatic surface representation of BdGF14c. The protein surface is colored according to its electrostatic potential: blue for positive and red for negative charge. **(E)** The precise position of the four critical amino acid identified by both Type-II functional divergence and positive selection. Each structure is rotated 90° to show different view sides of the protein.

The three-dimensional structures of seven BdGF14 proteins from *B. distachyon* were predicted by searching the SWISS-MODEL database and visualized using Pymol software. All *14-3-3* proteins displayed similar structural features; the representative BdGF14c is shown in Figure 4. The results showed that BdGF14c well-matched OsGF14c (PDB: 3AXY), with 97.02% identity (Figure 4A), in which the monomer consists of a bundle of nine anti-parallel α-helices (αA-αI, Figure 4B), and the dimer usually presents a characteristic cup-like shape with a large central channel containing two amphipathic binding grooves (Figures 4C,D). Four critical sites (1S, 2M, 96K, and 148S) were identified and shown to be associated with positive selection, as well as Type II functional divergence (Table S4). These sites are located on the N-terminal, αD- and αF-helices of the three-dimensional structure, respectively (Figure 4E). Multiple sequence alignment was performed to further investigate the location of the four critical sites in the protein sequences (Figure S8). Interestingly, all of these four sites were presented on the outer convex surface (Figure 4E). Further analysis showed that a large number of the identified sites from the Type II functional divergence (Table S4) were also located on the outer convex surface (data not shown).

### Analysis of promoter regions

The binding of transcription factors (TFs) to corresponding TF binding sites (TFBSs) upstream of target genes can regulate gene expression. Thus, the *cis*-acting elements in the 1500-bp region upstream of the translation initiation codon of the *14-3-3* gene family were identified using PlantCARE. In total, seven types of regulatory elements were found, four of which are strongly related to crucial physiological process, including light periods, developmental regulation, hormonal responses, and environmental responses (Table S7).

Among these elements, Sp1, G-box (Menkens et al., 1995), G-Box, Box 4 (Lois et al., 1989), and Box I (Arguello-Astorga and Herrera-Estrella, 1996) belonged to the light-responsive elements. Sp1 was shown to be the most abundant light-responsive element in the *14-3-3* gene family, with a mean number of 3.921 copies per gene, and it was much more abundant in lower plants (5.250) and monocots (6.780) than in dicots (1.229). The G-box was ranked as the second most abundant light-responsive element, followed by Box 4, G-Box, and Box I. These results suggest that *14-3-3* genes may function in photosynthesis and/or carbohydrate metabolism. Development-related elements were also identified, but their abundance showed no significant differences, except for CCGTCC-box (meristem development-related element), the abundance of which in lower plants and monocots was ~17.2- and 11.6-fold that in dicots, respectively. Phytohormones and other abiotic stress-responsive mechanisms also play crucial roles for defense against environmental stresses in plants. ABRE (Simpson et al., 2003), GARE-motif (Ogawa et al., 2003), TCA-element (Pastuglia et al., 1997), and TGA-element (Guilfoyle et al., 1993) are hormone-responsive elements. Among these elements, the ABRE element was found to be the most abundant (2.937), being related to the ABA signaling pathway, indicating that the expression of several *14-3-3* genes was induced by ABA-mediated signal transduction. Similarly, several elements were considered to be related to environmental stress, including MBS (Yamaguchi-Shinozaki and Shinozaki, 1993), ARE (Klotz and Lagrimini, 1996), HSE (Freitas and Bertolini, 2004), and TC-rich elements (Klotz and Lagrimini, 1996). Promoter analysis showed that the diversity of *cis*-acting regulatory elements in the upstream regions of *14-3-3* genes provided further support for the diversified functional roles of *14-3-3* genes in response to plant growth, development, and phytohormone and abiotic stresses.

### Expression profiles of *14-3-3* genes across different tissues and abiotic stresses in *B. distachyon*

**Figure 5:**
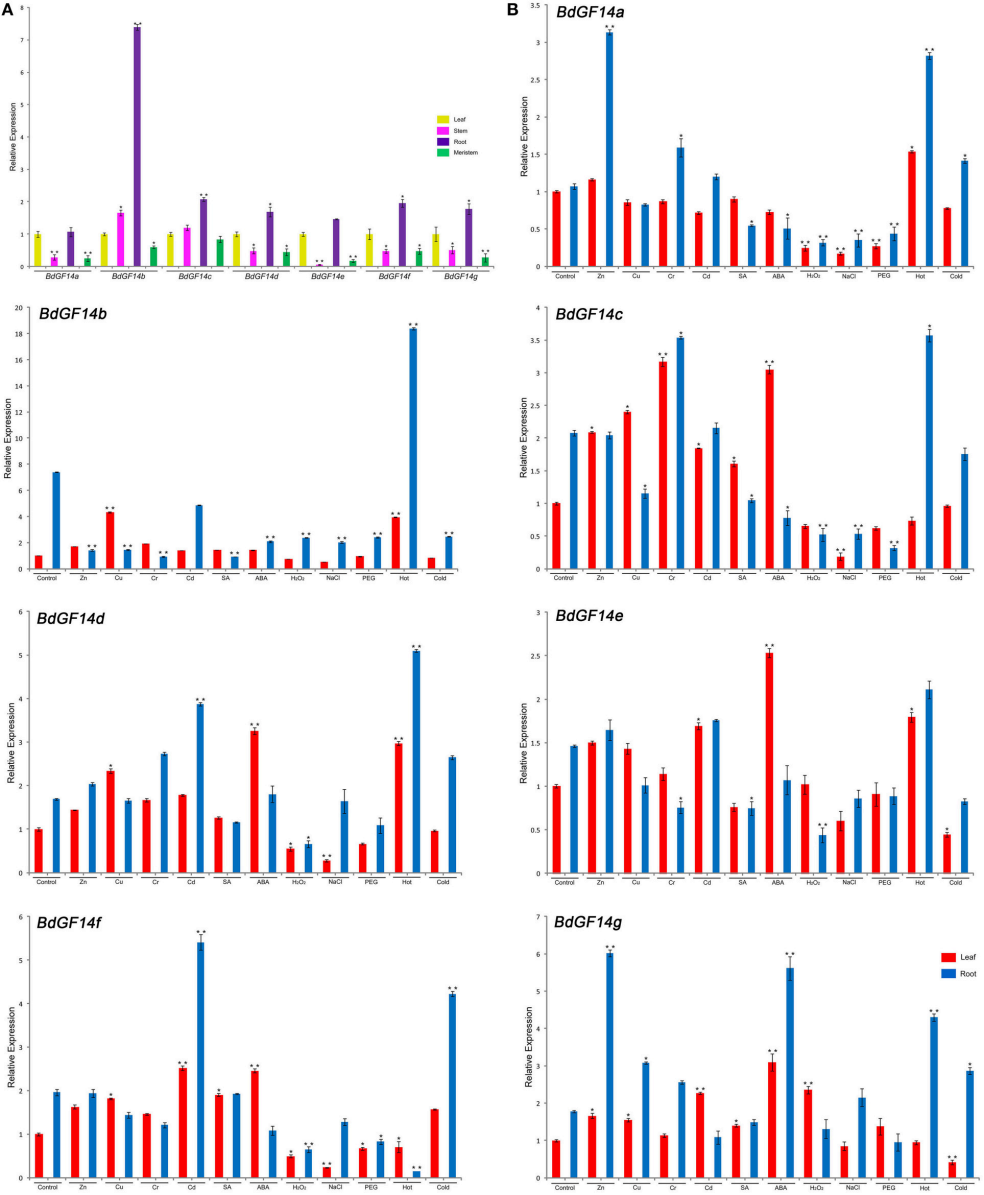
**Expression profiles of ***B. distachyon 14-3-3*** genes in tissues and under short time stress treatments**. **(A)** The expression profiles of seven *B. distachyon 14-3-3* genes in different tissues, including leaf, stem, root and meristem. **(B)** The remaining seven subfigures were expression profiles of seven *B. distachyon 14-3-3* genes under various abiotic stresses, including Zn^2+^, Cu^2+^, Cr^3+^, Cd^2+^, SA, ABA, H_2_O_2_, NaCl, PEG, Hot (42°C) and Cold (4°C). SA, salicylic acid; ABA, abscisic acid; PEG, polyethylene glycol. The expression levels of *14-3-3* genes in the control group were defined as 1.0. The leaf and root are separately analyzed, red color bar presents leaf, sky blue color bar presents root. Statistically significant differences between control group and treatment group were calculated by an independent Student's *t*-tests: ^*^P < 0.05, ^**^P < 0.01.

The transcriptional expression profiles of seven *BdGF14* genes from Bd21 in different organs and tissues (leaf, stem, root, and meristem) and abiotic stresses (salinity, drought, temperature, heavy metal, and phytohormone stresses) were analyzed by qRT-PCR. The results showed that all *BdGF14* genes were expressed in leaf, stem, root, and meristem, but had higher expression levels in root, particularly for *BdGF14b*, with 7.4-, 4.5-, and 12.3-fold higher expression than in leaf, stem, and meristem, respectively. In contrast, *BdGF14a* and *BdGF14e* in stem and meristem, and *BdGF14g* in meristem had lower expression (Figure 5A and Table S8).

The transcriptional expression changes of seven *BdGF14* genes in Bd21 seedling roots and leaves under various abiotic stresses were investigated, including osmotic [NaCl and polyethylene glycol (PEG)], temperature (hot: 42°C and cold: 4°C), heavy metal (Zn^2+^, Cu^2+^, Cr^3+^, and Cd^2+^), and phytohormone stresses [ABA, salicylic acid (SA), and H_2_O_2_]. Initially, we analyzed the expression profiles of *BdGF14* genes by applying 6 h treatments with salinity, drought, heavy metals, and phytohormones and 2 h treatments at the set temperatures. The morphological appearance under these treatments did not change significantly, except for H_2_O_2_ treatment, in which the seedling roots became brown after treatment (Table S9, Figure S9, S10).

In general, Seven *GdGF14* genes showed significant transcriptional expression changes under all stress treatments in both roots and leaves (Figure 5). In roots, *GdGF14* genes under different heavy metal stresses displayed upregulation or downregulation. Specifically, five genes (*GdGF14a, GdGF14c, GdGF14d, GdGF14e*, and *GdGF14f*) under Cd^2+^ stress, four (*GdGF14a, GdGF14d, GdGF14e*, and *GdGF14g*) under Zn^2+^ stress, and four (*GdGF14a, GdGF14c, GdGF14d*, and *GdGF14g*) under Cr^3+^ stress were upregulated. Under Cu^2+^ stress, only *BdGF14g* showed upregulated expression. Phytohormone and osmotic generally induced downregulated expression of *GdGF14* genes, but *BdGF14d* and *BdGF14g* under ABA stress and *BdGF14g* under NaCl stress were upregulated. Except for *GdGF14f*, which was downregulated, the other six genes were significantly upregulated under high-temperature stress. Four genes (*GdGF14a, GdGF14d, GdGF14f*, and *GdGF14g*) and three (*GdGF14b, GdGF14c*, and *GdGF14e*) under low-temperature stress were upregulated and downregulated, respectively.

*BdGF14* genes in leaves generally showed greater expression differences in response to abiotic stress. Except for *BdGF14a* showing downregulation under Cu^2+^, Cr^3+^, and Cd^2+^ stresses, the other genes were all upregulated under heavy metal stress. Compared with those in roots, more genes showed upregulated expression under phytohormone treatments, while similar expression patterns were found under NaCl and PEG treatments. In response to temperature stresses, four genes (*BdGF14a, BdGF14b, BdGF14d*, and *BdGF14e*) under a high temperature and only one gene (*BdGF14f*) under a low temperature were upregulated (Figure 5 and Table S8). Our results demonstrated that multiple abiotic stresses could induce the upregulated expression of individual *BdGF14* genes, including *BdGF14g* under seven treatments in roots and eight treatments in leaves, and four genes (*BdGF14b, BdGF14d, BdGF14e*, and *BdGF14f*) under seven treatments in leaves.

Based on the results above, five treatments (Cd^2+^, Cr^3+^, Cu^2+^, Zn^2+^, and ABA) generally induced significant upregulation of *GdGF14* genes. Thus, the dynamic transcriptional expression changes of seven *GdGF14* genes under long-term treatments with six time points (0, 6, 12, 24, and 48 h, and recovery 48 h) were further detected by qRT-PCR. The morphological observation results showed that, except for Zn^2+^ treatment, plant growth under stress treatments was significantly inhibited (Table S9 and Figure S10). After recovery for 48 h, almost no morphological changes were observed, except for Cd^2+^ treatment with minor changes. Interestingly, plants in the Zn^2+^ treatment group grew at a speed equivalent to those in the control group (Table S9 and Figure S10).

**Figure 6:**
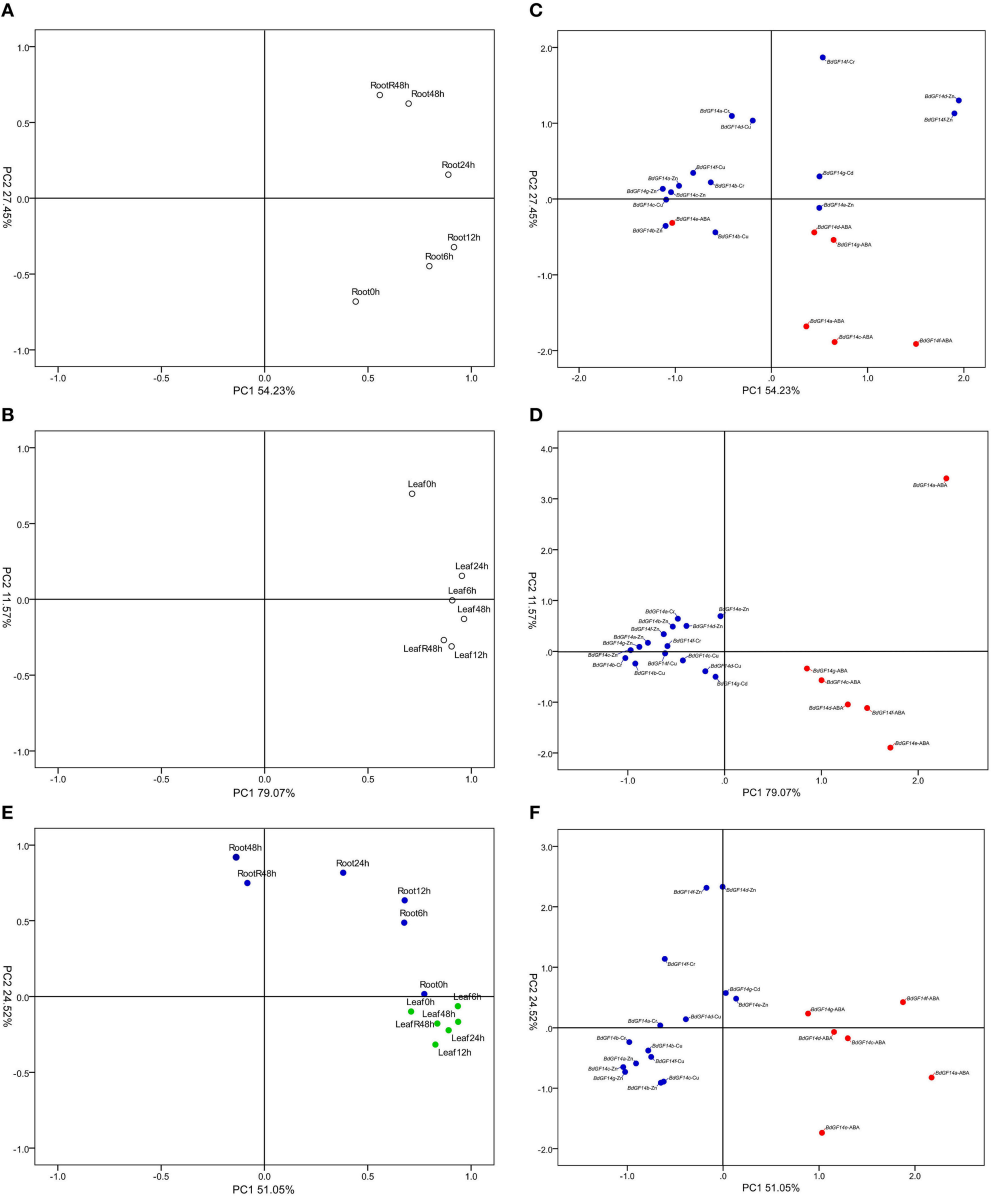
**PCA of ***B. distachyon 14-3-3*** genes in response to abiotic stresses**. **(A)** PCA of each treatment time point in the root. **(B)** PCA of each treatment time point in the leaf. **(C)** PCA of each treatment type in the root. **(D)** PCA of each treatment type in the leaf. **(E)** Integrative PCA of each treatment time point. **(F)** Integrative PCA of each treatment type. In the subfigure (**C,D,F**), blue color solid dots indicate heavy metal treatments, red color solid dots indicate ABA treatment. In the subfigure **(E)**, blue color solid dots indicate treatment in the root, green color solid dots indicate treatment in the leaf.

Principal component analysis (PCA) showed that the first 6 h induced larger expression changes of *BdGF14* genes than the subsequent periods in leaves (Figure 6A), while two periods (0–6 and 24–48 h) showed greater expression differences than the other three periods in roots (Figure 6B). Heavy metal (Cd^2+^, Cr^3+^, Cu^2+^, Zn^2+^) and phytohormone (ABA) treatments were clearly separated, indicating their distinct expression differences. The pattern in roots featured less close clustering than that in leaves (Figures 6C,D), indicating that roots had stronger responses against abiotic stresses than leaves. The integrative analysis results of roots and leaves showed similar patterns to the separate analyses mentioned above (Figures 6E,F).

**Figure 7:**
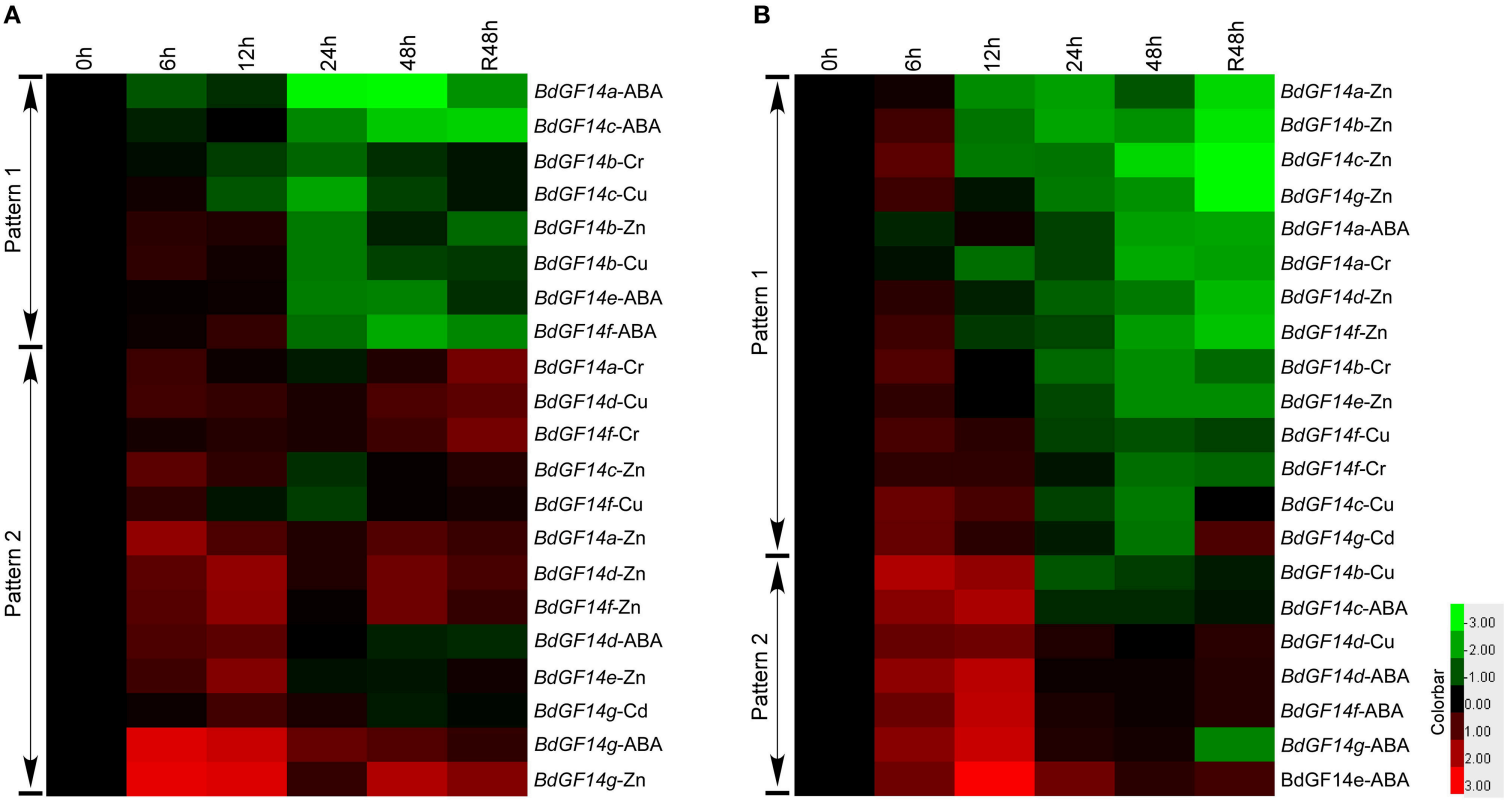
**Expression profiles of ***B. distachyon 14-3-3*** genes under long stress treatments**. **(A)** Gene expression profiles in the root. **(B)** Gene expression profiles in the leaf. Samples were harvested at 0, 6, 12, 24, 48, and recovery 48 h. The expression levels of *14-3-3* genes at 0 h were defined as 1.0 in both tissues.

Gene expression profiles revealed that all *BdGF14* genes in both roots and leaves showed similar expression patterns under five stress treatments, in which two distinct expression patterns, I and II, were present, corresponding to up-down and up-down-up expression trends from stress treatments to recovery, respectively (Figure 7). At 6 and 12 h after the five treatments, more genes showed significant upregulation in both roots and leaves, but all genes were downregulated at 24 h, and the lowest expression levels occurred at 48 h in leaves. However, the *BdGF14* genes at 48 h and recovery in roots displayed more significant upregulation than those in leaves.

## Discussion

In this paper, we present the first comprehensive survey on the molecular characterization, phylogenetic relationships, and abiotic stress responses of the *14-3-3* gene family in *B. distachyon*. Here, we focus on the structure, evolution, and function of plant *14-3-3* genes, combining our results and previous reports.

### Molecular characterization, phylogenetics, and evolutionary conservation of the *14-3-3* gene family in *B. distachyon*

Our results demonstrated that *BdGF14* genes, as well as other plant *14-3-3* genes, can be divided into five well-conserved subfamilies, but clear divergences occurred in different subfamilies, which is highly consistent with the results from *Arabidopsis* and rice (Chen et al., 2006). The genes in the same subfamily shared similar exon-intron organization and motif compositions, but greater structural differences were present between subfamilies (Figures S2–S6), suggesting the high structural conservation of plant *14-3-3* genes within a subfamily. The motif analysis results using the MEME online tool provide further evidence for the evolutionary conservation of the *14-3-3* genes in *B. distachyon* and other plant species, in which the former seven motifs from 5′ to 3′ were identical among 127 genes (Figures S5–S7).

Duplication events play critical roles in gene evolution, which is not only one of the primary driving forces in the evolution of genomic and genetic systems (Moore and Purugganan, 2003) but also a major mechanism for the establishment of new functions (Long and Langley, 1993). Segmental duplication was shown to be the predominant form by which the *14-3-3* gene family has expanded, which occurred about 10.1–58.6 MYA (Table 1). In *B. distachyon*, only one pair of segmental genes (*BdGF14e/BdGF14b*) was found, which was estimated to have arisen 45.6 MYA. The results from genome sequencing indicated that the divergence time of *B. distachyon* from wheat was 32–39 MYA, and 40–60 MYA from rice and sorghum (International Brachypodium Initiative, 2010). Thus, the segmental duplication event in the *B. distachyon 14-3-3* gene family occurred before the divergence from wheat and almost concurrently with the divergence from rice and sorghum.

Great variations can lead to gene diversification in biological functions (Zhu et al., 2015). The functional diversity of proteins was highly correlated with amino acid properties. We identified seven amino acid sites (1M, 2S, 96K, 148S, 236L, 237T, and 238E) as making important contributions to the functional diversity of *14-3-3* genes (Table 3, Tables S4, S6). Interestingly, the former four amino acids were mapped onto the outer convex surface of the three-dimensional structure of BdGF14c (Figure 4), and the remaining three were on the C-terminal (Figure S8). It is known that the inner walls of the central channel and the dimer interface in *14-3-3* proteins are formed by conserved residues, whereas the less conserved residues are located on the outer convex surface (Obsilova et al., 2014). In addition, the most flexible region of the *14-3-3* protein molecule is the C-terminal segment, which exhibits the highest sequence variability among *14-3-3* isoforms (Truong et al., 2002). Thus, we speculate that the diversity of amino acids at these sites may play roles in determining the functional divergence of *14-3-3* proteins.

### Expression and function of *14-3-3* genes in *B. distachyon*

*14-3-3* proteins show great functional diversity, being involved in a wide range of biological processes in plants, such as photosynthesis, primary metabolism, growth and cell division, hormone pathways, and abiotic and biotic stresses (de Boer et al., 2013). Results from cotton revealed that *14-3-3* proteins performed functions in cell elongation during the elongation phase of fiber development (Zhang et al., 2010). *GmGF14* genes abundantly expressed in embryos during early seed germination showed functions in soybean seed development (Li and Dhaubhadel, 2011), while *14-3-3* genes in *Medicago truncatula* were expressed almost equally in roots, stems, leaves, and flowers (Qin et al., 2016). The results from the present study indicated that the *14-3-3* genes are preferentially expressed in the roots and leaves (Figure 5A). During seedling growth phases, roots, and leaves are the most important organs necessary for nutrient absorption and photosynthesis, respectively. The abundant expression of *14-3-3* genes may contribute to the rapid vegetative growth of plant seedlings.

Considerable work has revealed that the *14-3-3* genes are involved in various abiotic stresses in different plant species, such as rice (Chen et al., 2006), *Populus* (Tian et al., 2015), common bean (Li et al., 2015), and *Hevea brasiliensis* (Yang et al., 2014). In particular, the presence of stress-related *cis*-elements in the promoter region of *14-3-3* genes could play important roles in responses to abiotic stresses. In the current study, significant expression changes of *BdGF14* genes in response to various abiotic stresses indicated their important roles in resisting such stresses. Particularly, high temperature induced the significant upregulation of most *BdGF14* genes, among which *BdGF14a, b, d*, and *e* were upregulated in both roots and leaves. However, *BdGF14* genes showed greater expression differences in these two organs under low-temperature stress: generally upregulation in roots and downregulation in leaves, but *BdGF14f* showed upregulated expression in both roots and leaves (Figure 5). At present, the mechanism of plant *14-3-3* gene function in high-temperature stress defense is still unclear. Considerable work has demonstrated that ABA/SA and Ca^2+^, representing ABA-dependent and calcium-dependent protein kinase (CDPK) signaling pathways, are involved in plant high-temperature stress responses (Mosser et al., 1990; Talanova and Titov, 1994; Lopez-Delgado et al., 1998). We speculate that *14-3-3* genes might also participate in these signaling pathways to defend against high-temperature stress. Except for the ABA-dependent signaling pathway that is considered as a critical signaling pathway involved in the low-temperature responses of plants (Roberts et al., 2002), functional analysis revealed that *RAC1A* (corresponding to *GRF3* in Figure 2) functions as a negative regulator in controlling the biosynthesis of ethylene to modulate constitutive freezing tolerance and cold acclimation in *Arabidopsis* (Catalá et al., 2014). However, investigation in maize roots found that *14-3-3* proteins participated in low-temperature and osmotic stress responses through binding to the plasma membrane H^+^-ATPase to activate the proton pump, leading to K^+^ influx and water uptake (Shanko et al., 2003).

ABA, a critical plant hormone, participates in a wide range of abiotic stresses, and regulates abiotic stress tolerance to cope with environmental stresses (Dong et al., 2015). Functional studies have indicated that *14-3-3* proteins interact with many TFs, such as the ABF family (Jakoby et al., 2002), WRKY (Rushton et al., 2010), and basic leucine zipper (bZIP; Sornaraj et al., 2016). These downstream TFs are involved in the ABA-dependent signaling pathway, important for plants in response to many abiotic stresses, such as phytohormone (ABA and SA), osmotic (salinity and drought), and temperature stresses (Rock, 2000; Dong et al., 2015). In the current study, we found that exogenous ABA and SA generally induced upregulation of *BdGF14* genes in leaves and downregulation in roots (Figures 5, 7). We speculate that plant *14-3-3* genes mainly participate in the ABA-dependent signaling pathway to cope with phytohormone stress by interacting with the downstream TFs.

Heavy metal contamination is a widespread and increasingly serious problem globally. The over-accumulation of reactive oxygen species (ROS) in cells is one of the most common consequences of heavy metal toxicity in plants, resulting from the interaction of heavy metals with electron transport activities (Shahid et al., 2014). We found that the majority of *BdGF14* genes were upregulated in leaves and downregulated in roots under heavy metal stresses (Figures 6, 7). One of the most common strategies for plants against heavy metal stresses is to activate the ROS-mediated mitogen-activated protein kinase (MAPK) cascade signaling pathway to scavenge extra ROS for detoxification (Jalmi and Sinha, 2015). *14-3-3* proteins, apart from binding to the downstream TFs, also interact with MAPK (Fritz et al., 2006). Thus, *14-3-3* genes perform their functions in response to heavy metal stress mainly through the ROS-mediated MAPK cascade signaling pathway.

NaCl, PEG, and H_2_O_2_ stresses generally induced significant downregulation of *BdGF14* genes (Figure 5). Previous reports showed that *PvGF14e* and *PvGF14h* genes in common bean and *ZmGF14-6* gene in maize (corresponding to *ZmGFc* in Figure 2) were downregulated under saline conditions and drought stress (Campo et al., 2012; Li et al., 2015), consistent with our results (Figure 5). A recent proteomic analysis revealed that *BdGF14b* and *BdGF14d* were downregulated under H_2_O_2_ stress in both roots and leaves (Bian et al., 2015). These *14-3-3* genes may be part of an adjustment in the perception of salinity and drought stresses (Talanova and Titov, 1994). Moreover, although phytohormone, osmotic, and temperature stresses mainly activate the ABA-dependent signaling pathway, these stresses can also induce the accumulation of ROS in plant cells plus the common downstream TFs. Thus, *14-3-3* genes could participate in different signaling pathways to cope with frequent and complex crosstalk caused by various abiotic stresses.

### Putative metabolic pathways of *14-3-3* genes involved in abiotic stresses

**Figure 8:**
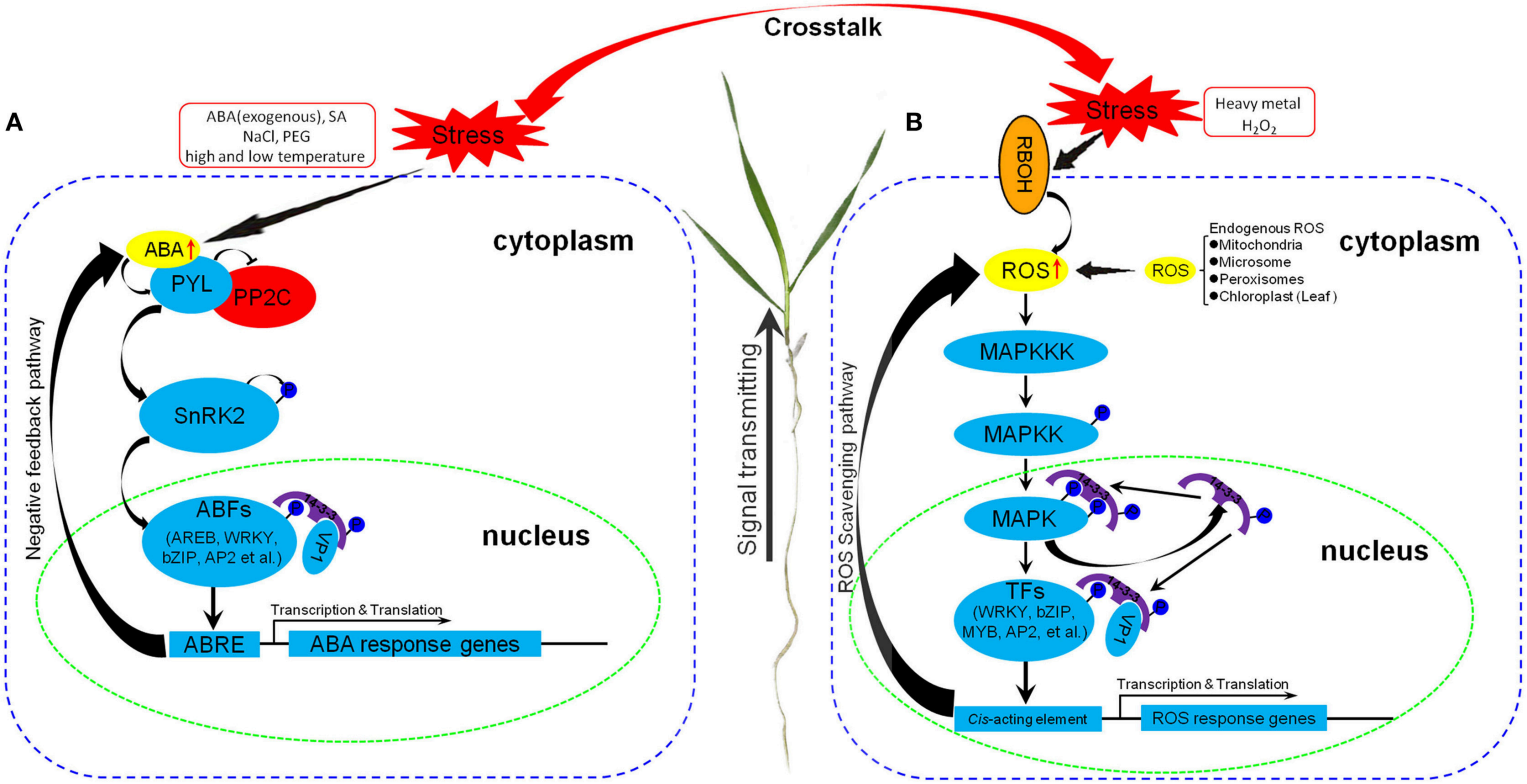
**Schematic representation of putative metabolic pathways of plant ***14-3-3*** genes involved in abiotic stresses**. **(A)** Metabolic pathway through ABA-dependent signaling pathway. **(B)** Metabolic pathway through ROS-mediated MAPK cascade signaling pathway. PYL, Pyrabactin resistance 1-like family; PP2C, type 2C protein phosphatase; SnRK2, Snf1-related protein kinase 2; ABF, ABRE-binding factor; AREB, ABRE-binding proteins; bZIP, Basic region/leucine zipper motif (bZIP) transcription factor; AP2, APETALA 2; VP1, Abscisic acid-VIVIPAROUS1; ABRE, ABA-responsive element; RBOH, Respiratory burst oxidase homolog; MAPK, Mitogen activated protein kinase; MAPKK, MAPK kinase; MAPKKK, MAPKK kinase; MYB, myeloblastosis family of transcription factor. Positive regulators are shown in sky-blue, negative regulators are shown in red, signal molecules are shown in yellow.

According to our results in combination with previous reports, we proposed the putative metabolic pathways of plant *14-3-3* genes in response to abiotic stresses, mainly involved in the ABA-dependent signaling pathway and/or ROS-mediated MAPK cascade signaling pathway (Figure 8). Abiotic stresses such as exogenous ABA, SA, NaCl, PEG, and high- and low-temperature stresses can induce the accumulation of ABA in cells, which subsequently triggers activation of the ABA-dependent signaling pathway and regulates the expression of downstream ABA-responsive genes against the stresses (Ng et al., 2014; Figure 8A). Meanwhile, the level of ROS in cells was increased by heavy metal stress, which triggered the MAPK cascade signaling pathway, and then initiated the expression of heavy metal stress response genes to scavenge the extra ROS for defense (Jalmi and Sinha, 2015) (Figure 8B). Under various abiotic stresses, plant *14-3-3* genes were upregulated and participated in these signaling pathways in response to abiotic stresses through interacting with TFs and regulating the expression of downstream stress response genes. Furthermore, various stresses result in frequent and complex crosstalk, and thus *14-3-3* genes are able to cope with multiple abiotic stresses through participating in different signaling pathways.

## Conclusion

The current study has demonstrated that plant *14-3-3* genes are highly conserved in individual subfamilies, but are divergent between different subfamilies. Segmental duplication seems to be the predominant form by which the *14-3-3* gene family has expanded, and all of the putative duplicated genes may postdate the monocot-dicot split. Functional divergence analysis revealed that the *14-3-3* gene family experienced radical shifts in cluster-specific amino acid properties, without significant changes in the evolutionary rate at certain amino acid sites. Transcriptional expression analysis of the *BdGF14* gene indicated that the *14-3-3* genes are significantly regulated by various abiotic stresses. Through participating in different signaling pathways and employing different strategies, *BdGF14* genes play important roles in the responses to multiple abiotic stresses. Meanwhile, frequent and complex crosstalk between different signaling pathways could occur under different adverse stresses. Individual *14-3-3* genes can cope with multiple abiotic stresses mainly through functioning in different signaling pathways. These results not only provide a better understanding of the evolutionary processes of *14-3-3* genes in plants but also offer a new insight into the molecular mechanisms of *14-3-3* genes in response to abiotic stresses in plants.

## Author contributions

YY and YH designed the study and provide guidance on the whole study. HC carried out the bioinformatic analysis, plant materials culture, qRT-PCR analysis, and drafted the manuscript. YX and LY participated in the qRT-PCR analysis. YB took parted in the plant materials culture. LW and SZ coordinated the study and elaborated on manuscript. All authors read and approved the final manuscript.

### Conflict of interest statement

The authors declare that the research was conducted in the absence of any commercial or financial relationships that could be construed as a potential conflict of interest.

## Supplementary material

Table S1 **Gene-specific primers for qRT-PCR analysis**.

Table S2 **The nomenclature, characteristics of ***14-3-3*** genes and their deduced proteins in 13 representative species**.

Table S3 **The parameters of conserved ***14-3-3*** domain from SMART and Pfam. (A)** The parameters of conserved *14-3-3* domain from SMART. **(B)** The parameters of conserved *14-3-3* domain from Pfam.

Table S4 **Type-II functional divergence sites identified between different subgroup pairs of ***14-3-3*** gene family**. Red color marked amino acids were identified in both Type-II functional divergence analysis and positive selection analysis. Amino acids listed in the table were matched to BdGF14c.

Table S5 **Tests for positive selection among codons of ***14-3-3*** genes using free-ratio and site models**. ^a^Number of parameters in the ω distribution.

Table S6 **Critical coevolution amino acids identified from coevolution analysis**.

Table S7 *****Cis***-element analysis of 1500-bp nucleotide sequences upstream of the translation initiation codon of ***14-3-3*** genes**.

Table S8 **Data of the relative expression of ***B. distachyon 14-3-3*** genes under abiotic stress treatments**.

Table S9 **Parameters of ***B. distachyon*** root length and plant size under control and abiotic stress treatments**. Dashes indicate no parameters collected.

Figure S1 **Double standard curves and dissolution curves of qRT-PCR**. The red standard curves represent the reference gene (*S-adenosylmethionine decarboxylase* gene), the blue standard curves represent the target genes. The dissolution curves of different genes are indicated.

Figure S2 **The number of ***14-3-3*** genes in individual species**. Cr, Chlamydomonas reinhardtii; Pp, Physcomitrella patens; Sm, Selaginella moellendorffii; Bd, Brachypodium distachyon; Os, Oryza sativa; Ta, Triticum aestivum; Si, Setaria italic; Zm, Zea mays; Sb, Sorghum bicolor; At, Arabidopsis thaliana; Gm, Glycine max; Sl, Solanum lycopersicum; Gr, Gossypium raimondii.

Figure S3 **Exon-intron organization of ***14-3-3*** genes**. The yellow boxes and gray lines represent exons and introns, respectively. The bold dark blue lines in the top indicate the 5′ upstream region (left) and the 3′ downstream region (right). The *14-3-3* genes were lined according to Phylogenetic tree construction.

Figure S4 **Statistic analysis in intron number. (A)** Statistic analysis based on subgroups. **(B)** Statistic analysis based on individual species.

Figure S5 **Schematic representation of conserved ***14-3-3*** protein motifs**. The schematic diagram was derived from MEME. The order of motifs in the Schematic representation was automatically generated by MEME according to scores.

Figure S6 **Schematic representation of amino acid motifs of ***14-3-3*** proteins**. Motif analysis was performed using MEME as described in the Methods. The gray solid lines represent the corresponding *14-3-3* proteins and their length. The different-colored boxes represent different motifs and their position and order in individual *14-3-3* protein sequence.

Figure S7 **Statistic analysis in motif number. (A)** Statistic analysis based on subgroups. **(B)** Statistic analysis based on individual species.

Figure S8 **Multiple sequence alignment of ***B. distachyon 14-3-3*** protein sequences. Eleven** *14-3-3* proteins consist of 7 *B. distachyon 14-3-3* proteins, 2 Arabidopsis *14-3-3* proteins, and 2 rice *14-3-3* proteins. Nine α helices are marked with red-colored solid rectangle, while N-terminal and C-terminal are marked with light blue-colored solid rectangle, respectively. Red boxes indicate the critical amino acids identified from functional divergence and positive selection, light blue box indicates the critical amino acids identified from coevolution, while green boxes indicate the critical amino acids identified only from positive selection.

Figure S9 **Images of ***B. distachyon*** plants under abiotic stress treatments for 6 or 2 h. (A)** Mild drought stress (PEG 6000). **(B)** Phytohormone stress (H_2_O_2_). **(C)** Phytohormone stress (SA). **(D)** Salinity stress (NaCl). **(E)** High temperature stress (42°C). **(F)** Low temperature stress (4°C). Bar scale is 5.0 cm.

Figure S10 **Images of Bd21 plants under abiotic stress treatments for 48 and 48 h recovery. (A)** Control group. **(B)** Heavy metal stress (Cu^2+^). **(C)** Heavy metal stress (Cr^3+^). **(D)** Heavy metal stress (Cd^2+^). **(E)** Heavy metal stress (Zn^2+^). **(F)** Phytohormone stress (ABA). Bar scale is 5.0 cm.

## Abbreviations

ABA: abscisic acid
ABRE: ABA-responsive element
ABF: ABRE-binding factor
AREB: ABRE-binding proteins
AP2: APETALA 2
bZip: Basic region/leucine zipper motif (bZIP) transcription factor
LRT: Likelihood ratio statistic
MW: Molecular weight
MAPK: Mitogen activated protein kinase
MAPKK: MAPK kinase
MAPKKK: MAPKK kinase
MYB: myeloblastosis family of transcription factor
PEG: polyethylene glycol
PYL: Pyrabactin resistance 1-like family
PP2C: type 2C protein phosphatase
PI: Isoelectric points
Qk: Posterior probability
RBOH: Respiratory burst oxidase homolog
SA: salicylic acid
SnRK2: Snf1-related protein kinase 2
TFBS: Transcription factor binding site
TF: Transcription factor
VP1: Abscisic acid-VIVIPAROUS1
WRKY: WRKY transcription factor.
