# Supplementary material for: Molecular Characterization of the 14-3-3 Gene Family in Brachypodium distachyon L. Reveals High Evolutionary Conservation and Diverse Responses to Abiotic Stresses
Source: Front Plant Sci. 2016 Jul 26;7:1099. doi: 10.3389/fpls.2016.01099 (PMC4960266; doi:10.3389/fpls.2016.01099)
Supplement: Table S4 — Type-II functional divergence sites identified between different subgroup pairs of 14-3-3 gene family. Red color marked amino acids were identified in both Type-II functional divergence analysis and positive selection analysis. Amino acids listed in the table were matched to BdGF14c. [file Table4.DOC]

**Table S4 | The Type-II functional divergence sites identified between different subgroup pairs of *14-3-3* gene family**

| **Group1** | **Group2** | **Type-II functional divergence sites** |
| --- | --- | --- |
| ε | non-ε-a | 1M, 2S, 5D, 6N, 13A, 24E, 27E, 30A, 31K, 32T, 34D, 49A, 56A, 57R, 60S, 78D, 79H, 81T, 82L, 83I, 88G, 92A, 96K, 99D, 100G, 103K, 105L, 107S, 113S, 115A, 116A, 119K, 122Y, 130H, 137K, 138A, 140A, 144E, 148S, 149T, 151V, 170I, 191K, 195L, 201D |
| ε | non-ε-b | none |
| ε | non-ε-c | 1M, 2S, 5D, 13A, 20E, 25Y, 27E, 31K, 32T, 49A, 64V, 77E, 78D, 79H, 81T, 82L, 88G, 90I, 92A, 97I, 99D, 100G, 104L, 105L, 106D, 107S, 113S, 116A, 119K, 122Y, 123L, 130H, 137K, 138A, 147E, 148S, 149T, 151V, 170I, 190D, 191K, 194N, 202E, 205S, 209T, 211T |
| ε | non-ε-d | none |
| non-ε-a | non-ε-b | 56A, 57R, 60S, 99D, 205S |
| non-ε-a | non-ε-c | 5D, 56A, 57R, 60S |
| non-ε-a | non-ε-d | 5D, 56A, 57R, 60S, 112M |
| non-ε-b | non-ε-c | none |
| non-ε-b | non-ε-d | none |
| non-ε-c | non-ε-d | none |

Note: Red color marked amino acids were identified in both Type-II functional divergence analysis and positive selection analysis. Amino acids listed in the table were matched to BdGF14c.
