# Supplementary material for: Molecular Characterization of the 14-3-3 Gene Family in Brachypodium distachyon L. Reveals High Evolutionary Conservation and Diverse Responses to Abiotic Stresses
Source: Front Plant Sci. 2016 Jul 26;7:1099. doi: 10.3389/fpls.2016.01099 (PMC4960266; doi:10.3389/fpls.2016.01099)
Supplement: Table S5 — Tests for positive selection among codons of 14-3-3 genes using free-ratio and site models. aNumber of parameters in the ω distribution. [file Table5.DOC]

| **Models** | ***np^a^*** | **Estimates of parameters** | **InL** | **Positively selected sites** |
| --- | --- | --- | --- | --- |
| Mf: free-ratio | 502 | None | -27325.319185 | None |
| M0: one-ratio | 253 | ω =0.03837 | -27797.471378 | None |
| M3: discrete | 257 | p_0_=0.62884 p_1_=0.24630 p_2_=0.12486  ω_1_ =0.00886 ω_2_=0.05021 ω_3_=0.19675 | -26866.726007 | None |
| M7: beta | 254 | p =0.47378 q =6.77453 | -26862.549359 | Not allowed |
| M8: beta&ɯ | 256 | p0 =0.99999 p=0.47380 q =6.77465  (p1 =0.00001 ) ω =1.00020 | -26862.551369 | None |

**Table S5 | Tests for positive selection among codons of *14-3-3* genes using free-ratio and site models**

Note: ^a^Number of parameters in the ω distribution
