# Supplementary material for: Molecular Characterization of the 14-3-3 Gene Family in Brachypodium distachyon L. Reveals High Evolutionary Conservation and Diverse Responses to Abiotic Stresses
Source: Front Plant Sci. 2016 Jul 26;7:1099. doi: 10.3389/fpls.2016.01099 (PMC4960266; doi:10.3389/fpls.2016.01099)
Supplement: Table S6 — Critical coevolution amino acids identified from coevolution analysis. [file Table6.DOC]

**Table S6 | The critical coevolution amino acids identified from coevolution analysis**

| **Protein name** | **Coevolution amino acids** |
| --- | --- |
| BdGF14c | 236L, 237T, 238E |
