# Supplementary material for: Molecular Characterization of the 14-3-3 Gene Family in Brachypodium distachyon L. Reveals High Evolutionary Conservation and Diverse Responses to Abiotic Stresses
Source: Front Plant Sci. 2016 Jul 26;7:1099. doi: 10.3389/fpls.2016.01099 (PMC4960266; doi:10.3389/fpls.2016.01099)
Supplement: Table S9 — Parameters of B. distachyon root length and plant size under control and abiotic stress treatments. Dashes indicate no parameters collected. [file Table9.DOC]

**Table S9 | The parameter of *B. distachyon* root length and plant size under control and abiotic stress treatment**

| **Samples** | **Treatment time** | | | | | | | | | | | |
| --- | --- | --- | --- | --- | --- | --- | --- | --- | --- | --- | --- | --- |
|  | **0h** | | **6h** | | **12h** | | **24h** | | **48h** | | **R48h** | |
|  | **Root Length/(cm)** | **Plant Size/(cm)** | **Root Length/(cm)** | **Plant Size/(cm)** | **Root Length/(cm)** | **Plant Size/(cm)** | **Root Length/(cm)** | **Plant Size/(cm)** | **Root Length/(cm)** | **Plant Size/(cm)** | **Root Length/(cm)** | **Plant Size/(cm)** |
| Control | 7.6±0.28 | 13.7±0.32 | 7.5±0.23 | 13.7±0.25 | 7.6±0.03 | 14.0±0.84 | 7.5±0.33 | 14.1±0.17 | 8.0±0.32 | 15.3±0.37 | 9.5±0.63 | 17.0±1.31 |
| SA | 9.5±0.81 | 16.4±1.38 | 9.3±1.09 | 16.3±1.46 | ___ | ___ | ___ | ___ | ___ | ___ | ___ | ___ |
| H_2_O_2_ | 4.3±0.32 | 9.2±0.31 | 4.5±0.40 | 9.5±0.45 | ___ | ___ | ___ | ___ | ___ | ___ | ___ | ___ |
| PEG | 4.2±0.09 | 9.7±0.41 | 4.2±0.11 | 9.8±0.55 | ___ | ___ | ___ | ___ | ___ | ___ | ___ | ___ |
| NaCl | 11.4±1.33 | 19.0±1.14 | 12.2±1.32 | 20.5±1.33 | ___ | ___ | ___ | ___ | ___ | ___ | ___ | ___ |
| Hot | 13.2±0.18 | 19.3±0.67 | 12.3±0.78 | 18.5±0.84 | ___ | ___ | ___ | ___ | ___ | ___ | ___ | ___ |
| Cold | 10.1±0.46 | 16.5±0.57 | 11.1±1.59 | 17.9±1.61 | ___ | ___ | ___ | ___ | ___ | ___ | ___ | ___ |
| ABA | 12.5±1.64 | 20.4±2.00 | 12.8±2.02 | 20.6±0.23 | 12.6±1.73 | 20.4±2.10 | 12.3±1.16 | 20.2±1.55 | 12.7±1.89 | 20.5±2.31 | 12.8±1.81 | 20.6±2.13 |
| Zn | 12.3±0.83 | 18.8±0.66 | 12.8±1.07 | 19.8±0.76 | 13.0±0.69 | 19.8±0.68 | 12.8±0.93 | 19.9±0.58 | 13.0±0.83 | 20.6±0.38 | 14.8±0.71 | 22.5±0.81 |
| Cu | 12.1±1.59 | 19.2±1.99 | 11.9±1.57 | 19.1±2.10 | 12.5±1.72 | 19.4±2.31 | 12.1±1.64 | 19.3±2.29 | 12.2±1.72 | 19.4±2.34 | 12.2±1.44 | 19.2±2.09 |
| Cr | 9.1±0.79 | 15.6±1.10 | 9.2±0.71 | 15.9±0.90 | 9.4±0.72 | 16.0±0.95 | 8.9±0.79 | 15.6±1.09 | 9.3±0.73 | 16.0±0.96 | 9.2±0.66 | 15.9±0.78 |
| Cd | 6.5±0.45 | 13.0±0.60 | 6.5±0.18 | 12.8±0.27 | 6.5±0.14 | 12.8±0.17 | 6.7±0.23 | 13.1±0.18 | 6.5±0.14 | 12.9±0.13 | 7.1±0.92 | 13.8±0.17 |

Note: Dashes indicate no parameters collected.
