# Supplementary figures and images for: Molecular Characterization of the 14-3-3 Gene Family in Brachypodium distachyon L. Reveals High Evolutionary Conservation and Diverse Responses to Abiotic Stresses
Source: Front Plant Sci. 2016 Jul 26;7:1099. doi: 10.3389/fpls.2016.01099 (PMC4960266; doi:10.3389/fpls.2016.01099)

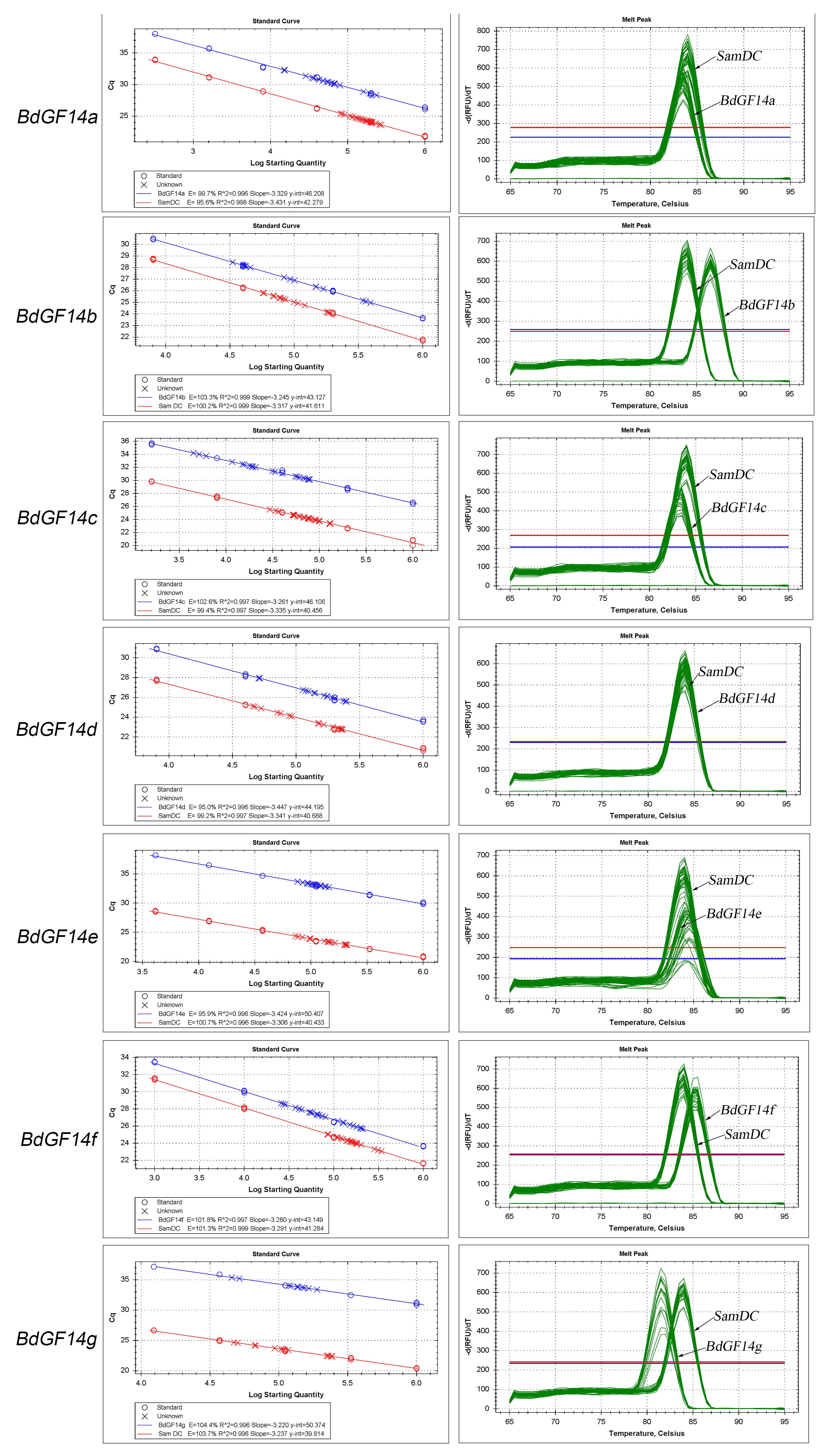

Supplement: Figure S1 — Double standard curves and dissolution curves of qRT-PCR. The red standard curves represent the reference gene (S-adenosylmethionine decarboxylase gene), the blue standard curves represent the target genes. The dissolution curves of different genes are indicated. [file Image1.JPEG]

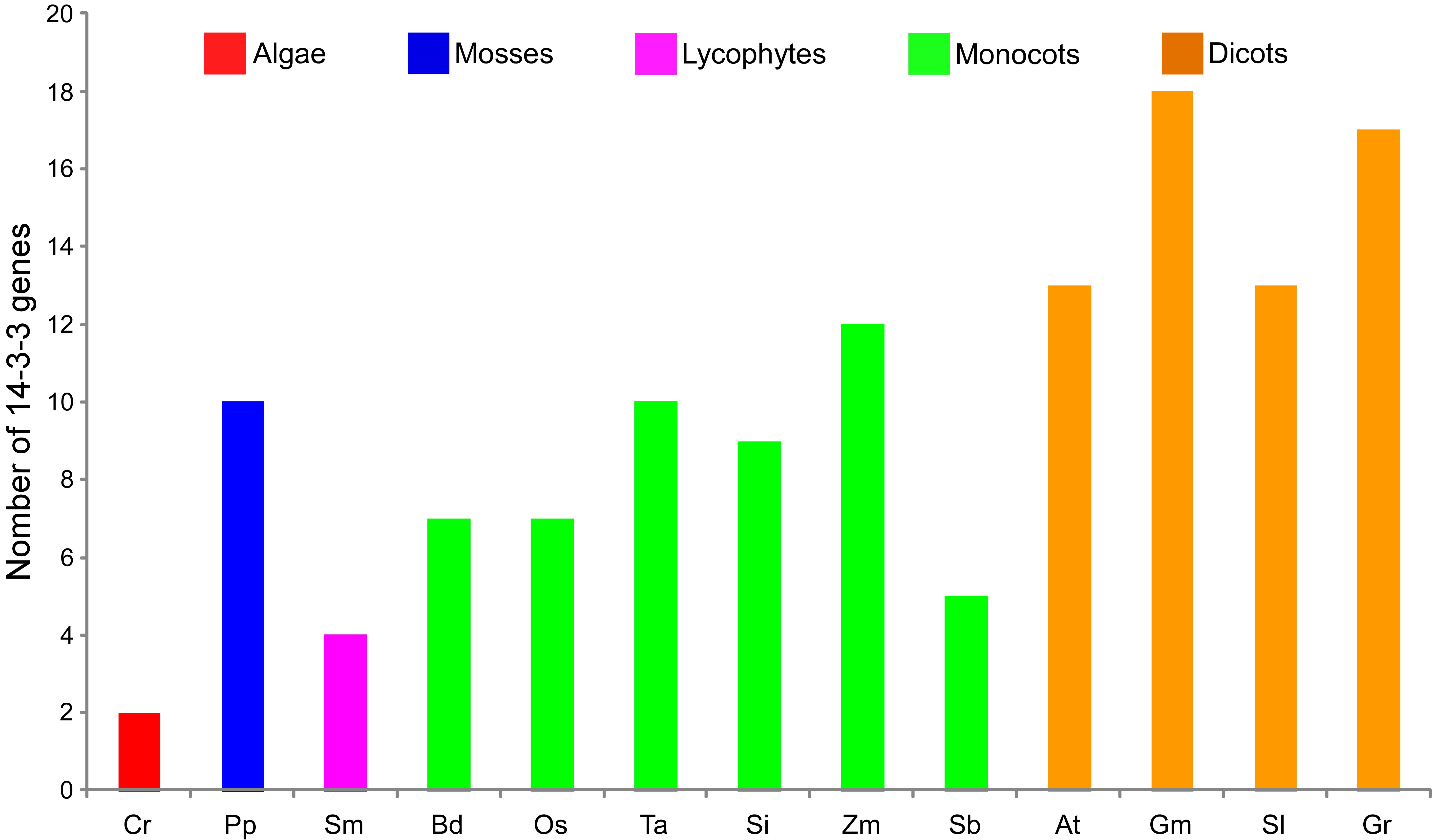

Supplement: Figure S2 — The number of 14-3-3 genes in individual species. Cr, Chlamydomonas reinhardtii; Pp, Physcomitrella patens; Sm, Selaginella moellendorffii; Bd, Brachypodium distachyon; Os, Oryza sativa; Ta, Triticum aestivum; Si, Setaria italic; Zm, Zea mays; Sb, Sorghum bicolor; At, Arabidopsis thaliana; Gm, Glycine max; Sl, Solanum lycopersicum; Gr, Gossypium raimondii. [file Image2.JPEG]

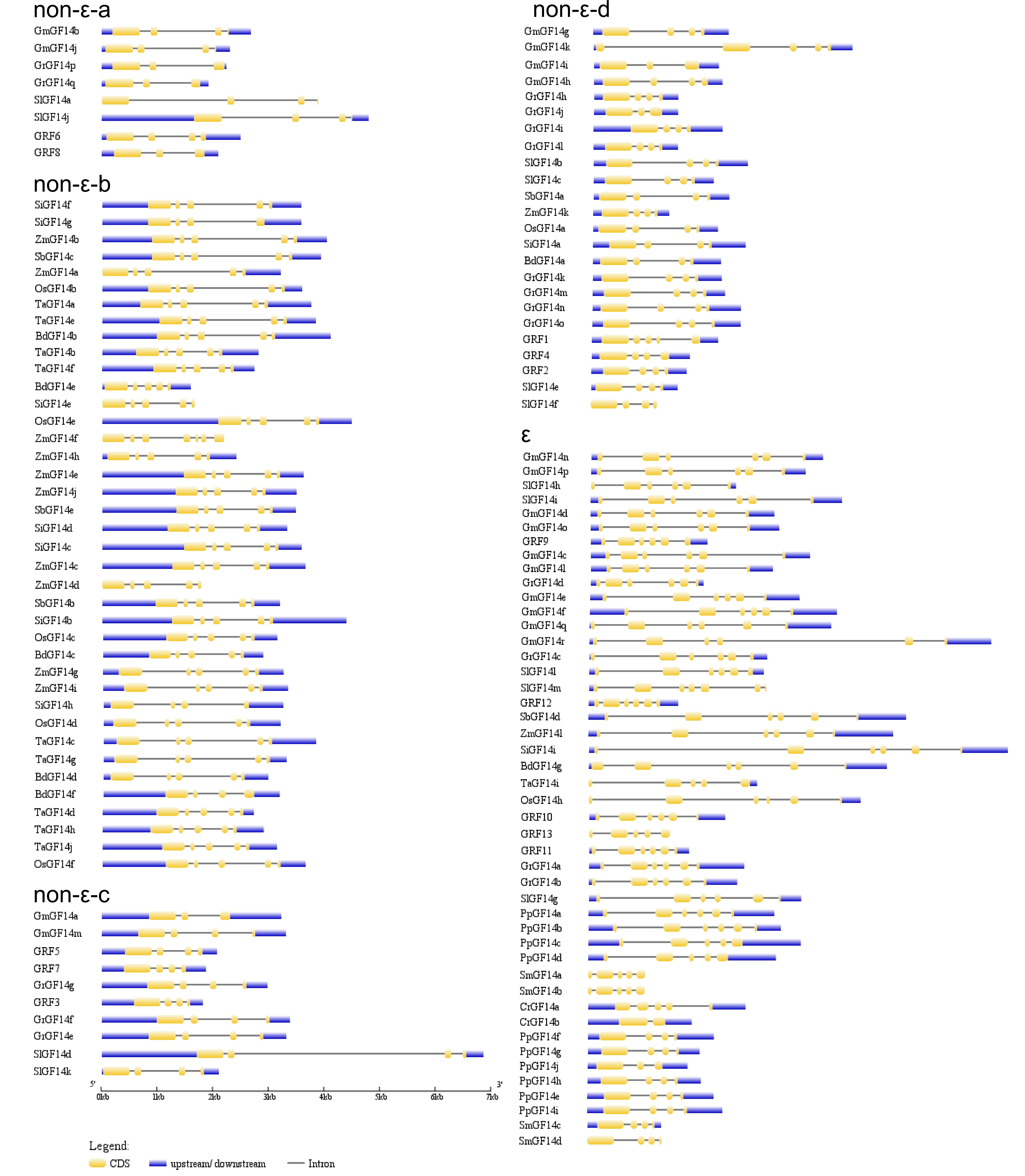

Supplement: Figure S3 — Exon-intron organization of 14-3-3 genes. The yellow boxes and gray lines represent exons and introns, respectively. The bold dark blue lines in the top indicate the 5′ upstream region (left) and the 3′ downstream region (right). The 14-3-3 genes were lined according to Phylogenetic tree construction. [file Image3.JPEG]

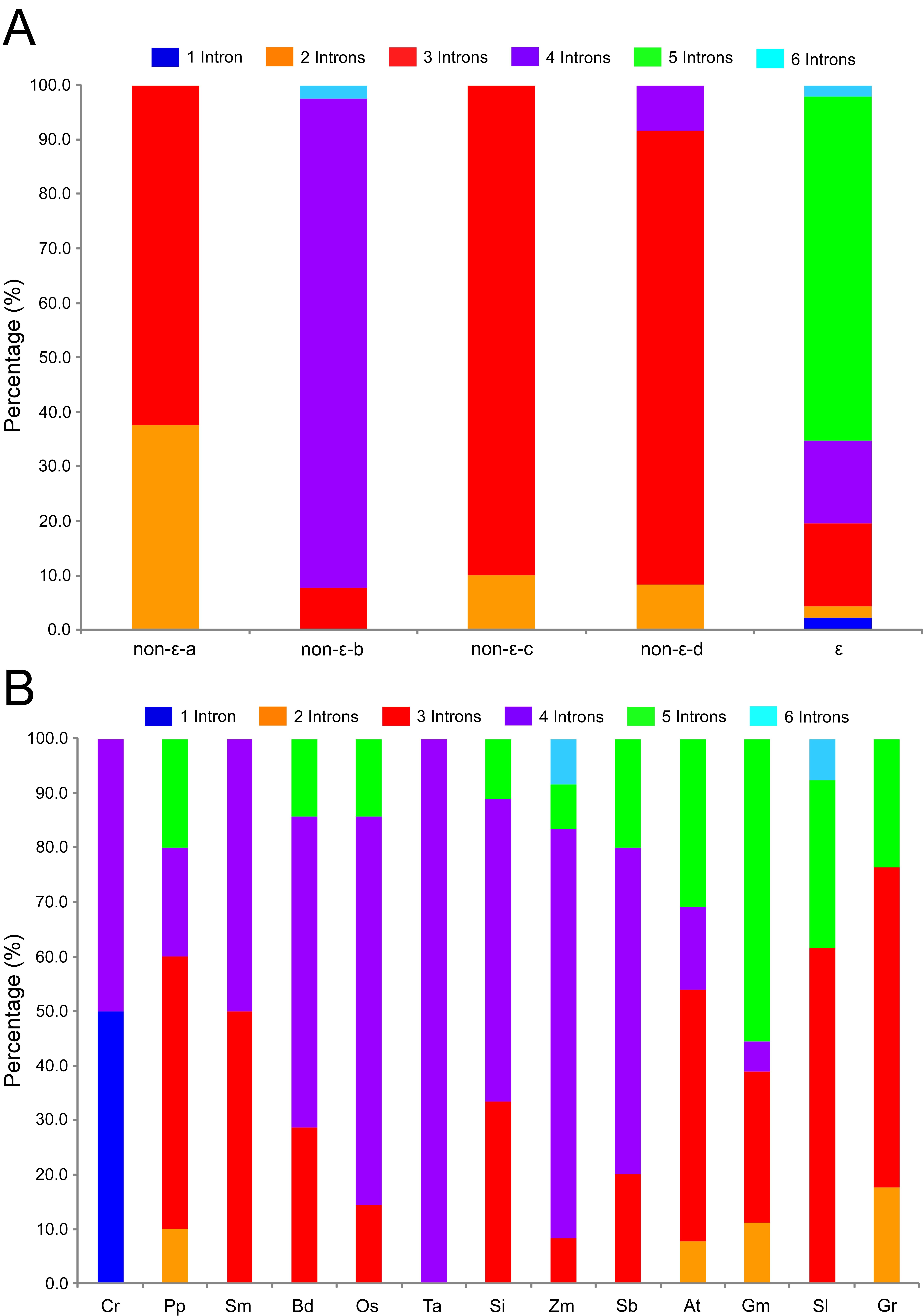

Supplement: Figure S4 — Statistic analysis in intron number. (A) Statistic analysis based on subgroups. (B) Statistic analysis based on individual species. [file Image4.JPEG]

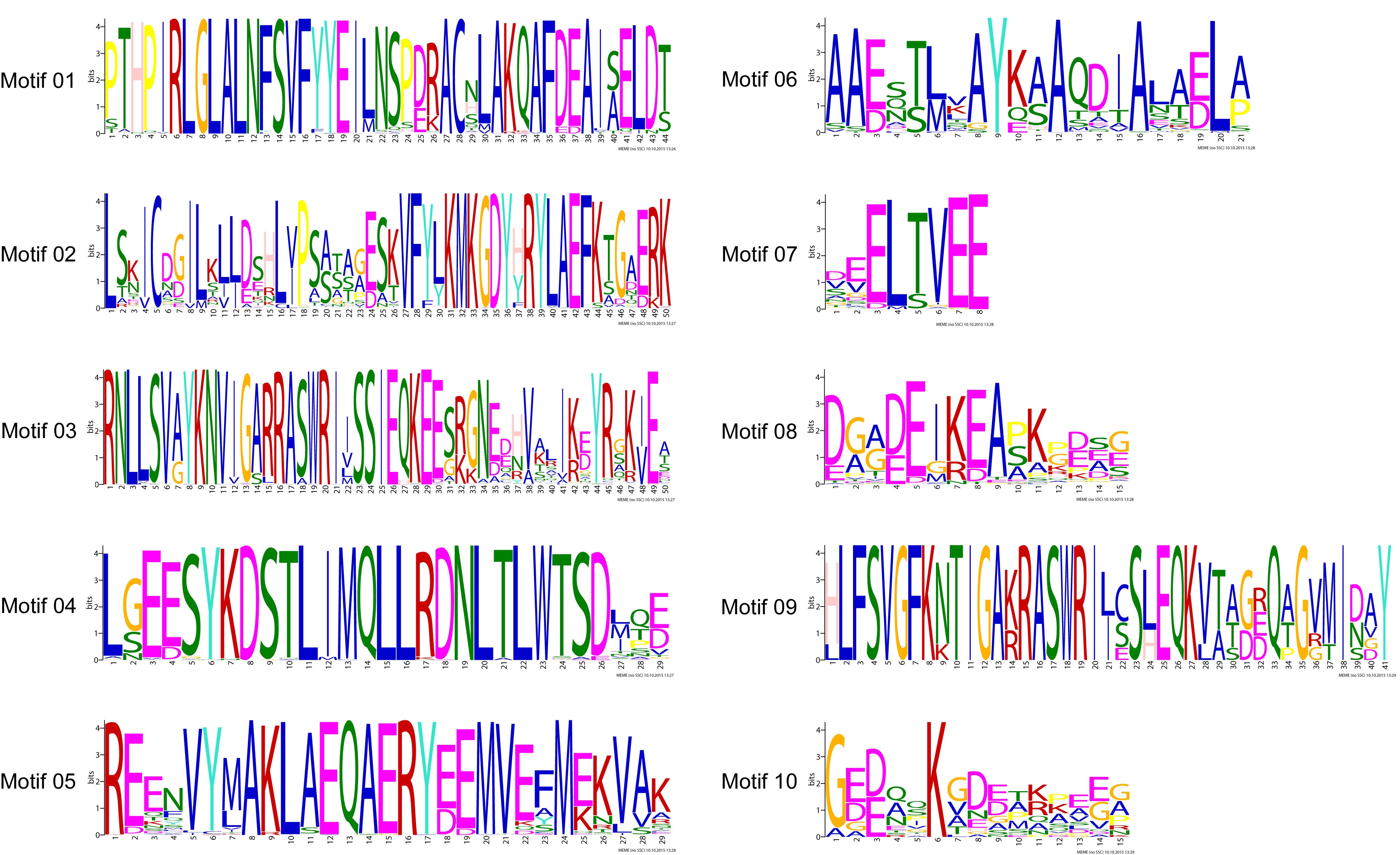

Supplement: Figure S5 — Schematic representation of conserved 14-3-3 protein motifs. The schematic diagram was derived from MEME. The order of motifs in the Schematic representation was automatically generated by MEME according to scores. [file Image5.JPEG]

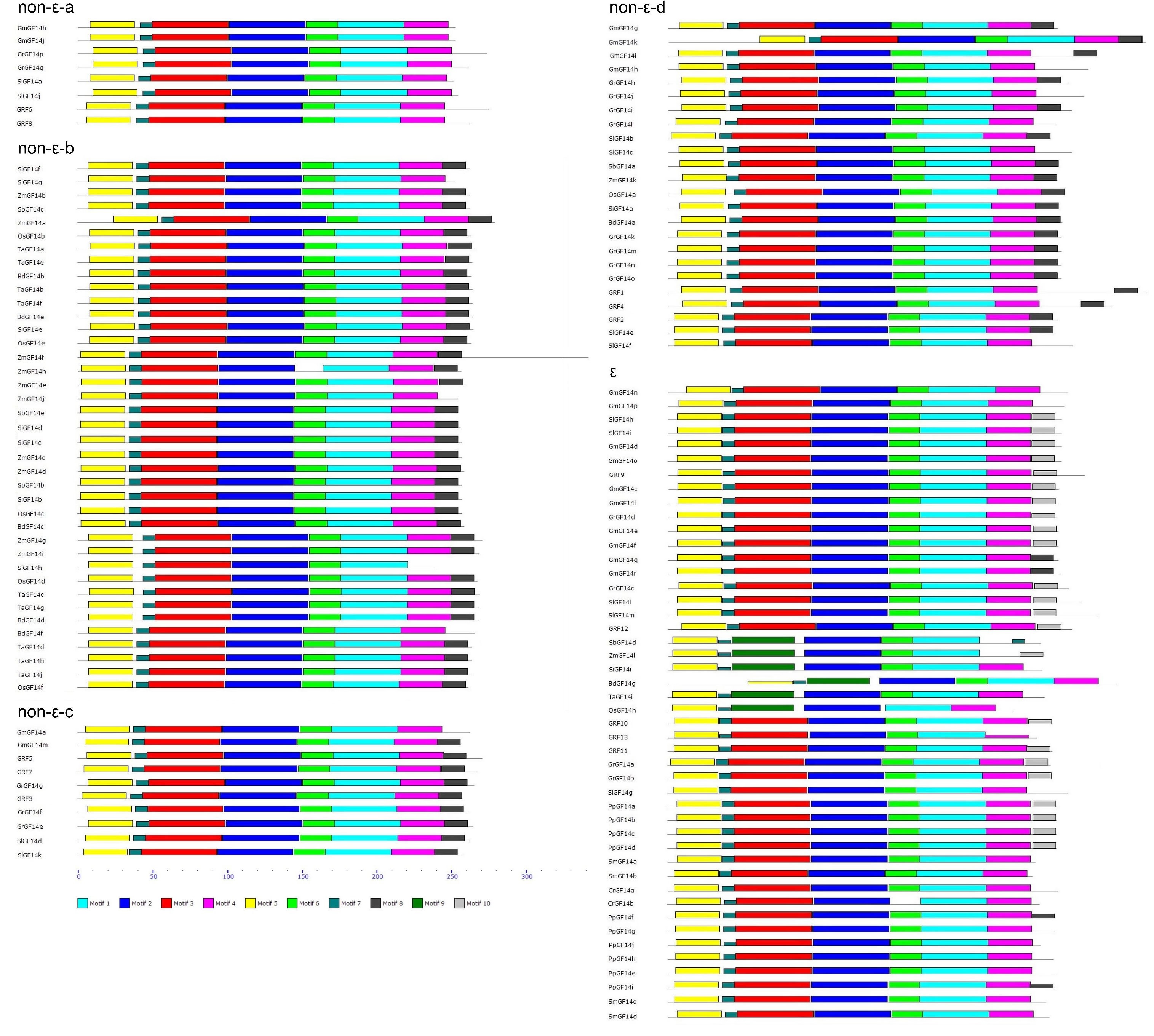

Supplement: Figure S6 — Schematic representation of amino acid motifs of 14-3-3 proteins. Motif analysis was performed using MEME as described in the Methods. The gray solid lines represent the corresponding 14-3-3 proteins and their length. The different-colored boxes represent different motifs and their position and order in individual 14-3-3 protein sequence. [file Image6.JPEG]

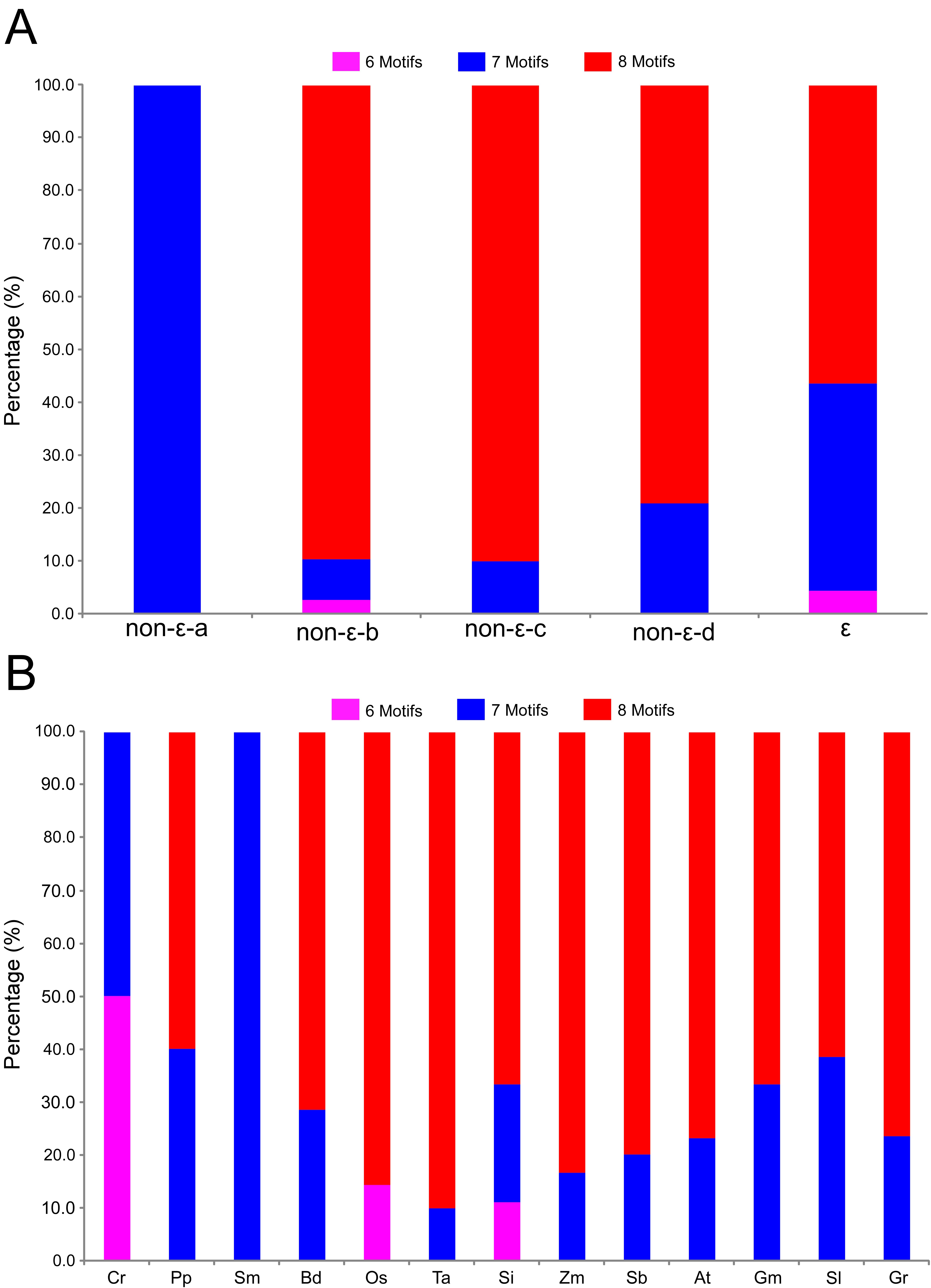

Supplement: Figure S7 — Statistic analysis in motif number. (A) Statistic analysis based on subgroups. (B) Statistic analysis based on individual species. [file Image7.JPEG]

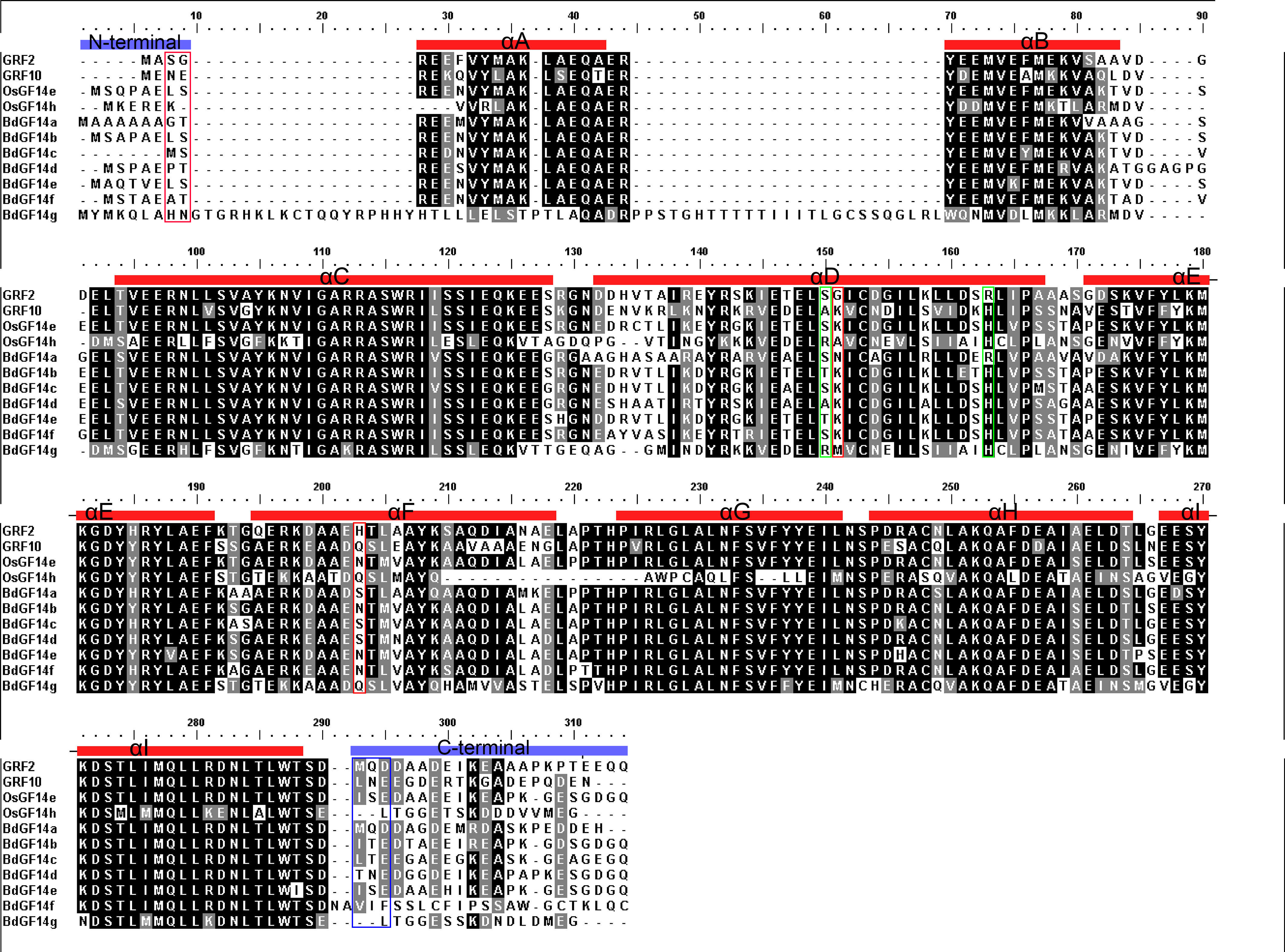

Supplement: Figure S8 — Multiple sequence alignment of B. distachyon 14-3-3 protein sequences. Eleven 14-3-3 proteins consist of 7 B. distachyon 14-3-3 proteins, 2 Arabidopsis 14-3-3 proteins, and 2 rice 14-3-3 proteins. Nine α helices are marked with red-colored solid rectangle, while N-terminal and C-terminal are marked with light blue-colored solid rectangle, respectively. Red boxes indicate the critical amino acids identified from functional divergence and positive selection, light blue box indicates the critical amino acids identified from coevolution, while green boxes indicate the critical amino acids identified only from positive selection. [file Image8.JPEG]

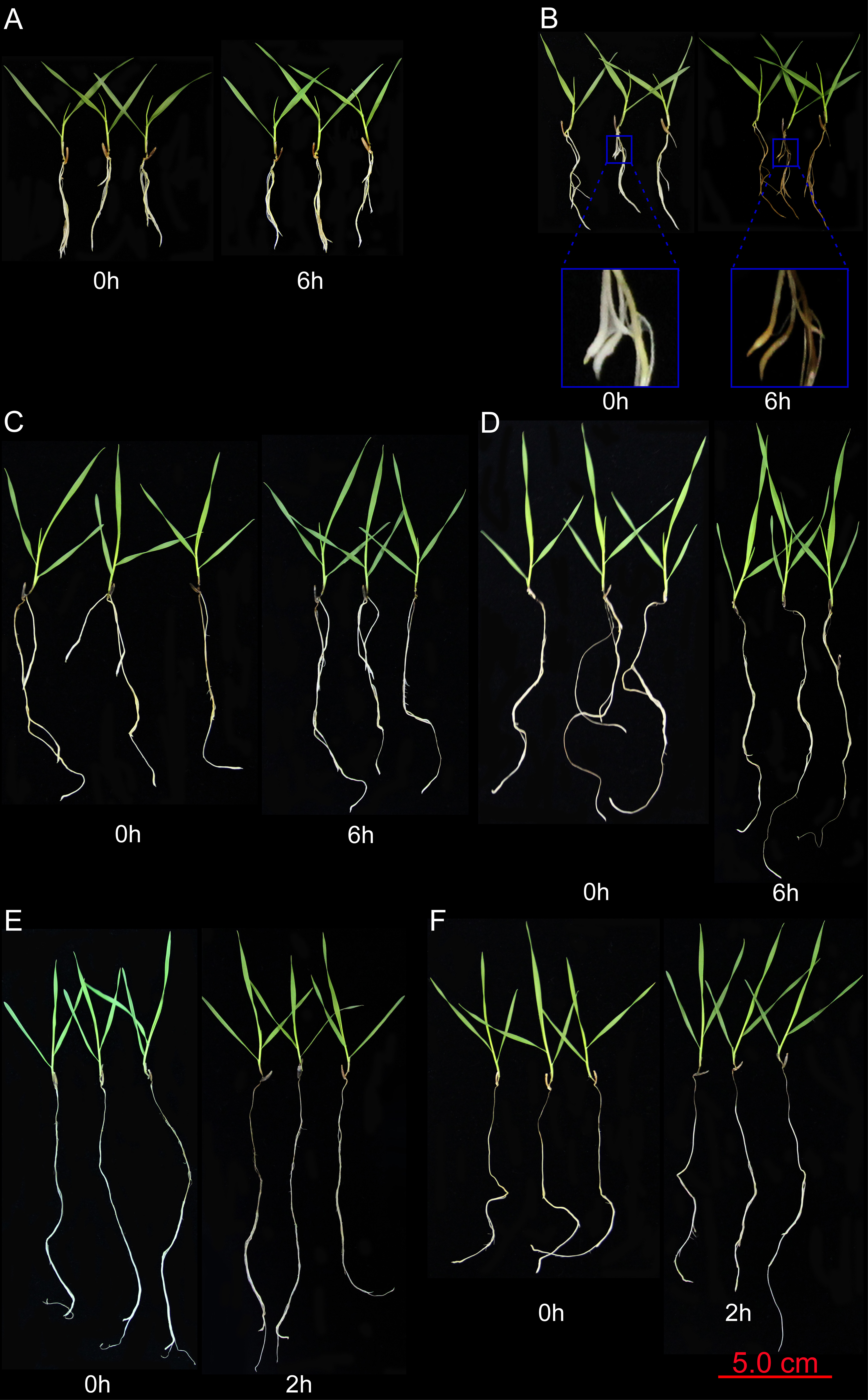

Supplement: Figure S9 — Images of B. distachyon plants under abiotic stress treatments for 6 or 2 h. (A) Mild drought stress (PEG 6000). (B) Phytohormone stress (H2O2). (C) Phytohormone stress (SA). (D) Salinity stress (NaCl). (E) High temperature stress (42°C). (F) Low temperature stress (4°C). Bar scale is 5.0 cm. [file Image9.JPEG]

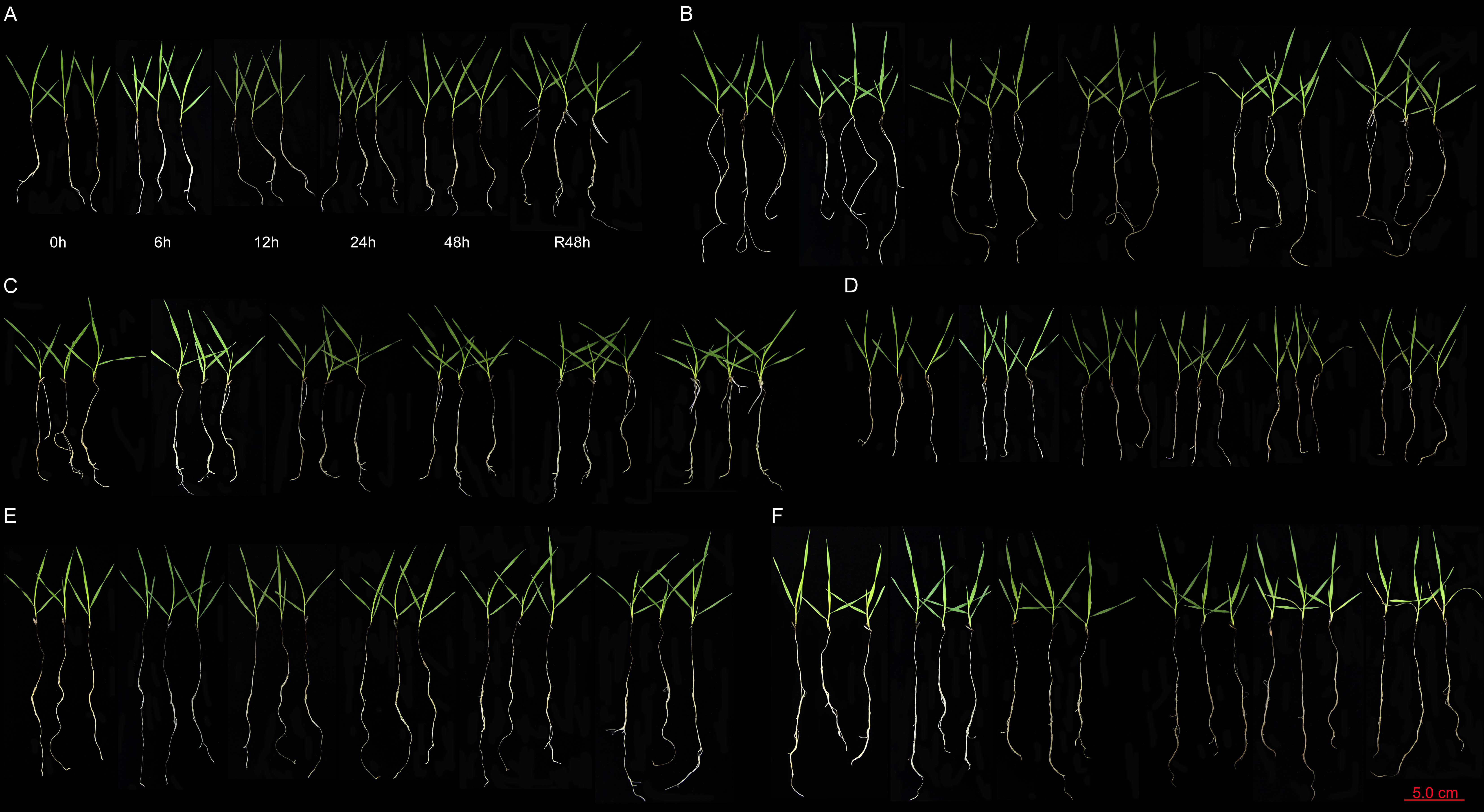

Supplement: Figure S10 — Images of Bd21 plants under abiotic stress treatments for 48 and 48 h recovery. (A) Control group. (B) Heavy metal stress (Cu2+). (C) Heavy metal stress (Cr3+). (D) Heavy metal stress (Cd2+). (E) Heavy metal stress (Zn2+). (F) Phytohormone stress (ABA). Bar scale is 5.0 cm. [file Image10.JPEG]
